# A transformer-based deep learning algorithm for diagnosing spinal infections on axial non-contrast computed tomography images: a dual-center retrospective study

**DOI:** 10.7717/peerj.21340

**Published:** 2026-06-11

**Authors:** Dongdong Yu, Zhenting Hu, Kai Song, Wenxin Lu, Jingfeng Xu, Jiali Zheng, Yongjian Kang, Yuan Hong, Bin Chen

**Affiliations:** 1 Department of Orthopedic Surgery, First Affiliated Hospital, Zhejiang University School of Medicine, Hangzhou, Zhe Jiang, China; 2 Department of Electrical and Computer Engineering, Stevens Institute of Technology, Hoboken, New Jersey, United States of America; 3 The First Affiliated Hospital of Henan Medical University, Xin Xiang, He Nan, China; 4 Department of Radiology, First Affiliated Hospital, Zhejiang University School of Medicine, Hangzhou, Zhejiang, China; 5 College of Mathematical Medicine, Zhejiang Normal University, Jinhua, China

**Keywords:** Spinal infections, Deep learning, Computed tomography

## Abstract

**Background:**

Spinal infections are rare but serious conditions requiring timely diagnosis. Non-contrast computed tomography (CT) is widely used and may incidentally reveal spinal abnormalities; however, subtle infectious findings are often missed, especially on axial images without sagittal reconstruction.

**Objectives:**

To investigate a deep learning approach for diagnosing primary spinal infections using non-contrast CT images.

**Methods:**

This retrospective dual-center study included 157 patients with primary spinal infection. A Swin Transformer model was developed using non-contrast CT slices. Patients from the primary center (*n* = 127) were randomly split 7:3 into training and internal validation sets. An independent external cohort (*n* = 30) from a second center was used for external validation. Per-slice diagnostic performance was assessed using the area under the receiver operating characteristic curve (AUC), area under the precision-recall curve (AUPRC), sensitivity, specificity, accuracy, positive predictive value (PPV), negative predictive value (NPV), and F1-score, with a probability threshold ≥0.5 determined by the Youden index. Model performance was compared with that of two radiologists.

**Results:**

The Swin Transformer model demonstrated excellent per-slice diagnostic performance. In the internal validation set, the model achieved an AUC of 0.979, sensitivity of 96.2%, specificity of 90.5%, and accuracy of 89.4%. In the external cohort, similar results were obtained: AUC 0.989, sensitivity 98.2%, specificity 98.3%, and accuracy 98.3%. The deep learning model significantly outperformed both radiologists in AUC and sensitivity across cohorts (all *P* < 0.05). With artificial intelligence (AI) assistance, both radiologists showed substantial improvements in diagnostic performance and efficiency in both internal and external validation (all *P* < 0.05). The model’s reading time was markedly shorter than that of unassisted radiologists, and AI assistance reduced radiologists’ reading time (*P* < 0.001). Presence of spinal epidural abscess and pathogen type significantly influenced diagnostic outcomes (*P* < 0.001).

**Conclusions:**

This Swin Transformer-based deep learning model achieves high diagnostic accuracy for detecting spinal infections on axial non-contrast CT images, with performance comparable to or exceeding that of musculoskeletal radiologists. By enhancing radiologists’ sensitivity and reducing reading time, the model shows promise as a clinical decision support tool to reduce missed diagnoses, particularly in emergency or resource-limited settings where magnetic resonance imaging (MRI) is unavailable. Its robust performance on external validation supports generalizability and lays the foundation for future multicenter prospective studies and extension to other spinal pathologies.

## Introduction

Spinal infections are rare and difficult to diagnose and treat, accounting for 2%–7% of all musculoskeletal infections. They can lead to pathological fractures, neurological impairment, spinal deformities, paralysis, and even death in advanced stages. The incidence of spinal infections is rising annually due to an aging population, an increasing number of immunocompromised patients, and antibiotic misuse (Raghavan & Palestro, 2023; Baryeh et al., 2022). Moreover, the clinical manifestations, laboratory findings, and imaging features of spondylodiscitis are often atypical, increasing the risk of both misdiagnosis and missed diagnosis (Encarnación-Santos et al., 2024; Crombé et al., 2024). Given the importance of timely diagnosis and treatment, the development of a safe, rapid, non-invasive, and accurate diagnostic method would greatly assist clinicians in making informed decisions (Tsantes et al., 2020; Duarte & Vaccaro, 2013).

Currently, a positive microbiological culture from a percutaneous or open biopsy remains the gold standard for diagnosing spondylodiscitis. However, this method is limited by a prolonged turnaround time and a high false-negative rate, often due to prior antibiotic use or inadequate sampling. Moreover, the procedure is invasive and carries multiple risks and contraindications (Duarte & Vaccaro, 2013). As a result, non-contrast computed tomography (CT) is widely used for the incidental detection of spinal infections. Although CT has lower sensitivity and specificity than magnetic resonance imaging (MRI), it is cost-effective and readily available, making it an essential alternative for patients who cannot undergo MRI. In addition, CT-guided percutaneous aspiration biopsy, combined with three-dimensional CT reconstructions, remains a valuable tool for obtaining pathological tissue (Crombé et al., 2024; Expert et al., 2021). Incidental spinal infections detected on routine chest or abdominal CT scans are frequently underreported, delaying early diagnosis—a problem particularly pronounced in economically disadvantaged areas (Müller et al., 2008; Zhu et al., 2024; Babic & Simpfendorfer, 2017; Bhise et al., 2017). This is largely attributed to the limited familiarity of thoracic and abdominal radiologists with spinal pathology (Carberry et al., 2013).

Artificial intelligence and deep learning have been increasingly applied to identify atypical and rare diseases across a wide range of imaging modalities. Various computer-aided detection (CAD) systems have demonstrated promising preliminary results (Haug & Drazen, 2023). Doyle et al. (2020) showed that machine learning improved the detection of patients with non-tuberculous mycobacterial lung disease by nearly a thousand-fold, achieving an area under the curve (AUC) of 0.94. Similarly, Motohashi et al. (2024) developed a deep learning-based CAD model for the automated detection of osteolytic bone metastases in the thoracolumbar spine. These advances highlight the potential of deep learning to enhance image interpretation and support radiologists in diagnosing spinal infections.

In recent years, artificial intelligence (AI) has been increasingly applied to the imaging diagnosis of spinal infections. However, existing studies have primarily focused on classification tasks—distinguishing among infection types (*e.g.*, tuberculous *vs.* pyogenic spondylitis) (Kim et al., 2018; Nijiati et al., 2025; Yang et al., 2025) or differentiating infection from other spinal pathologies (*e.g.*, metastatic disease, osteoporotic fractures) (Liu et al., 2025). Most of these studies are based on MRI and predominantly employ convolutional neural network (CNN) architectures (Mo et al., 2026), achieving performance comparable to that of radiologists in these specific classification tasks (Yang et al., 2025; Aljawadi et al., 2019). Nevertheless, this research paradigm has two critical limitations. First, it primarily addresses the differentiation of known infection types, with little emphasis on the screening and *de novo* detection of spinal infections, particularly on more accessible modalities such as non-contrast CT (Mo et al., 2026). Second, from a model architecture perspective, Transformer-based models—which excel at capturing long-range dependencies and may be particularly suited for identifying complex, spatially distributed lesion patterns—remain largely unexplored in this domain. Notably, prior studies have largely focused on diagnostic classification once an infection is suspected, rather than on early detection or screening in routine imaging. For instance, earlier CNN-based approaches have shown utility in distinguishing vertebral osteomyelitis from other pathologies on MRI, yet they remain dependent on high-contrast imaging and are not tailored for screening in more accessible modalities such as non-contrast CT (Mo et al., 2026; Müller et al., 2008). Similarly, other spine-related AI work has emphasized segmentation, fracture detection, or tumor classification, often leveraging CNNs or hybrid models, but has seldom addressed the specific challenge of identifying infectious foci within heterogeneous spinal anatomy on CT (Nijiati et al., 2025). Detecting spondylodiscitis itself is challenging, as images often contain coexisting degenerative changes (*e.g.*, osteophytes, disc herniation) and exhibit substantial anatomical variation across individuals (Liebl et al., 2021; Tang et al., 2024). These factors complicate the identification of lesions with diverse imaging features at different spinal levels. To address this gap in screening-oriented tasks, the present study aims to develop a Swin Transformer-based deep learning model for the automatic detection of spinal infections on axial non-contrast CT images.

However, the current diagnostic paradigm for spinal infections—whether based on MRI or CT—remains largely focused on binary patient-level classification (infected *vs.* non-infected). While essential for screening, this approach provides no spatial information regarding which vertebral levels are involved, where to biopsy, or how to stage the disease. As highlighted in a comprehensive review by Crombé et al. (2024) imaging in spondylodiscitis serves four key roles: (i) initial detection, (ii) differential diagnosis, (iii) disease staging, and (iv) guidance for percutaneous interventions such as CT-guided biopsy. Each of these tasks requires precise lesion localization—knowledge that a binary patient-level label cannot provide. Consequently, there is an urgent unmet need for computational tools that not only identify infected patients but also localize infectious foci at the slice level on routine non-contrast CT, thereby directly augmenting radiologists’ diagnostic workflow and enabling targeted interventions.

Therefore, this study aimed to develop and externally validate a Swin Transformer-based deep learning model for the per-axial CT slice detection of spinal infections. We further evaluated its impact on radiologists’ diagnostic accuracy and reading time and performed a complementary patient-level analysis to confirm its screening performance. This dual-level evaluation framework is designed to address both the population screening and precision localization demands of real-world clinical practice.

## Patients and Methods Study Design and Participants

A retrospective analysis was conducted on patients with spinal infection who were admitted to The First Affiliated Hospital, Zhejiang University School of Medicine between November 2020 and August 2023. The inclusion criteria were as follows: a definitive diagnosis of spinal infection confirmed by postoperative pathological examination or etiological testing, availability of preoperative non-contrast CT images, and complete clinical data. The exclusion criteria were: (1) Unclear postoperative pathological diagnosis; (2) Concurrent tumors or other immune-related diseases; (3) Incomplete or unclear imaging data; (4) Incomplete clinical data. A total of 127 patients with confirmed spinal infection were ultimately enrolled in the study. Furthermore, to further validate the generalizability of the model, an external validation cohort comprising 30 patients with spinal infection from The First Affiliated Hospital of Henan Medical University was introduced. Data collection for the primary cohort was carried out from March 2024 to July 2024, which involved retrieving eligible patients’ preoperative non-contrast CT scans and basic clinical information from the Picture Archiving and Communication System (PACS). Data for the external validation set were collected on January 20, 2026. Patients with prior spinal instrumentation, postoperative implant-associated infections, or any foreign body in the spinal column were excluded from the outset to restrict the study cohort to primary vertebral osteomyelitis or spondylodiscitis only. This was an a priori design choice to ensure pathophysiological homogeneity and to avoid the confounding effects of metallic artifact and distinct infectious mechanisms.

A total of 127 patients from the primary center were enrolled and randomly divided into a training set and an internal validation set at an approximate ratio of 7:3 using a computer-generated random number sequence. No additional validation set was carved out from the training data; instead, model development and hyperparameter tuning were performed exclusively on the training set, and the internal validation set was held out entirely until final evaluation to ensure an unbiased estimate of model performance. To further assess generalizability, an independent external validation cohort of 30 patients was collected from a different institution, acquired using different CT scanners and imaging protocols. This external cohort was not used in any phase of model development and was analyzed only once after the final model was locked.

**Figure 1:**
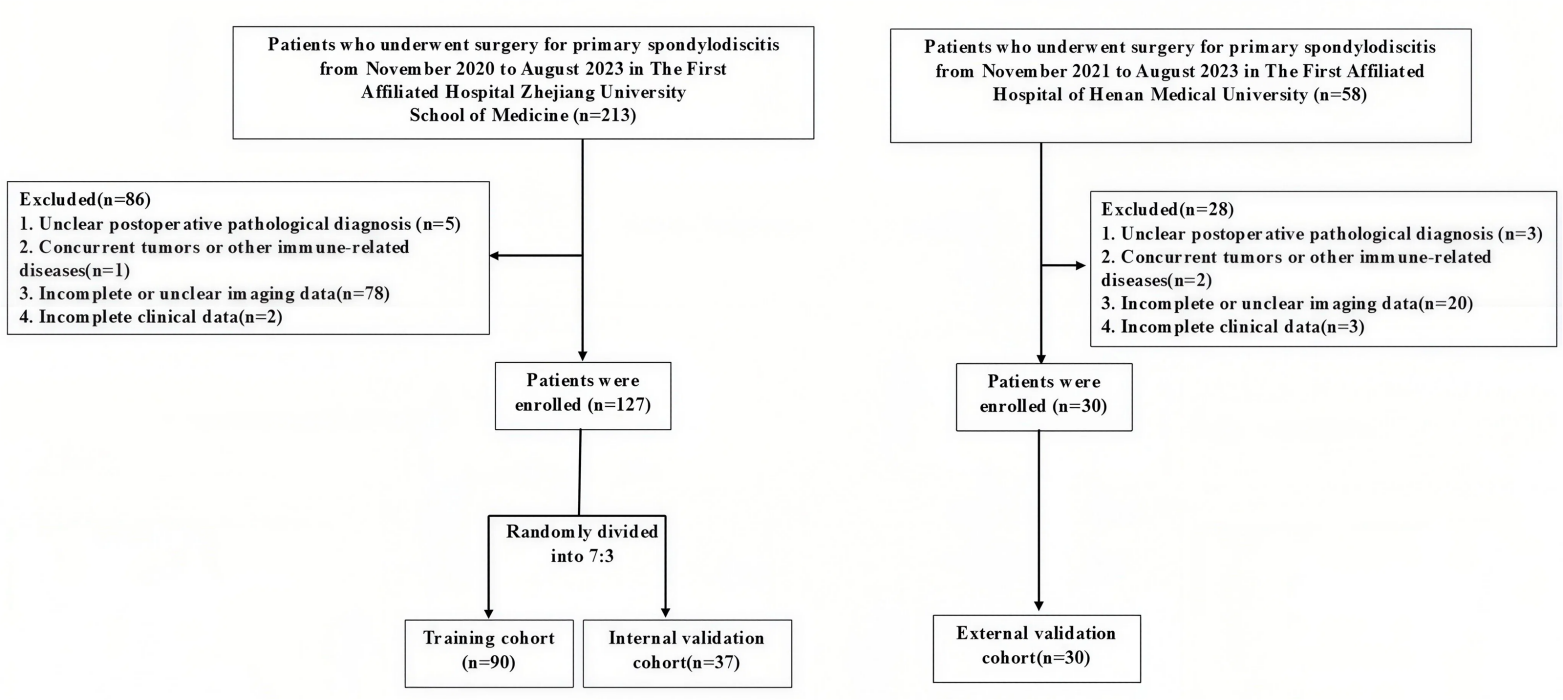
Flowchart of patient selection and cohort division for the study on primary spondylodiscitis.

All procedures involving human participants in this study were conducted in accordance with the ethical standards of the institutional research committee, the 1964 Declaration of Helsinki, and its subsequent amendments. The study protocol was approved by the Ethics Committee of The First Affiliated Hospital, Zhejiang University School of Medicine (No. 20240304) and by the Ethics Committee of The First Affiliated Hospital of Henan Medical University (No. 202609). Informed consent was waived by the Institutional Review Board due to the retrospective nature of the study. Pathogen identification was performed *via* standard bacterial culture or next-generation sequencing (NGS) of the specimens. The patient screening process is illustrated in Fig. 1.

### Imaging acquisition

Non-contrast computed tomography (CT) scans were acquired using multi-detector scanners from various institutions. All scans were retrieved in DICOM format and anonymized prior to analysis. A total of 157 patients with serial non-contrast CT images were included in this study; the examinations comprised pulmonary, abdominal, and spinal studies. We analyzed large field-of-view axial obtained from routine non-contrast chest, abdominal, or thoracoabdominal CT protocols. No dedicated spine-specific reformations (*e.g.*, sagittal or coronal reconstructions) were used, in order to reflect real-world clinical conditions where spinal findings are often encountered incidentally and dedicated spine imaging is not routinely performed. To ensure uniformity, all images were analyzed in the transverse plane with a slice thickness ≤1.25 mm (range: 0.625–1.25 mm), and no contrast administration or sagittal reconstruction was performed. For each patient, every axial slice from the entire non-contrast series was included, irrespective of whether the slice contained evidence of spinal infection. This approach was deliberately chosen to closely simulate real-world clinical practice, where radiologists must interpret the full imaging study and distinguish a small number of positive slices from a large background of negative slices. In total, 2,142 positive and 16,563 negative CT cross-sectional slices were obtained from the 157 patients.

### Image annotation and slice classification

All selected images were normalized to a window width of 300–500 HU and a window level of 40–60 HU to optimize soft tissue contrast, then resized to 512 × 512 pixels and uploaded to a dedicated annotation platform.

Under the general supervision of two board- certified musculoskeletal radiologists (each with >10 years of experience), a radiologic technician manually delineated the boundaries of each spinal infection lesion on a slice-by-slice basis, capturing the size, location, and morphological characteristics of the affected areas (*e.g.*, vertebral body, intervertebral disc, paravertebral soft tissue, epidural abscess).

An axial CT slice was classified as positive if it contained any portion of a manually segmented lesion; otherwise, it was classified as negative. All slices from the complete non- contrast CT series of each patient were included, regardless of the presence of infection, to reflect real-world clinical conditions.

To ensure annotation accuracy, all segmentations were independently reviewed by the second musculoskeletal radiologist, and any discrepancies were resolved through consensus. Both annotators were blinded to clinical, pathological, and microbiological information during the entire annotation process.

Following this protocol, a total of 2,142 positive slices and 16,563 negative slices were obtained from the 157 patients included in the study (127 internal + 30 external). The number of axial slices per patient varied depending on the anatomical coverage of the scan. Detailed statistics—including mean, standard deviation, and range of slice counts for both the internal and external validation cohorts—are provided in Table S1.

### Patient level diagnostic evaluation

In addition to the primary slice level analysis, we assessed model performance at the patient level. The evaluation cohort included all 37 infected patients from the internal validation set and 127 additional non infected control patients who underwent non contrast CT for non-spinal indications during the same period, with no evidence of spinal infection confirmed by clinical and imaging follow up (≥6 months). Patient level classification was defined as positive if any axial slice from the entire CT series was predicted as infected (probability ≥0.5).

### Deep learning model development

**Figure 2:**
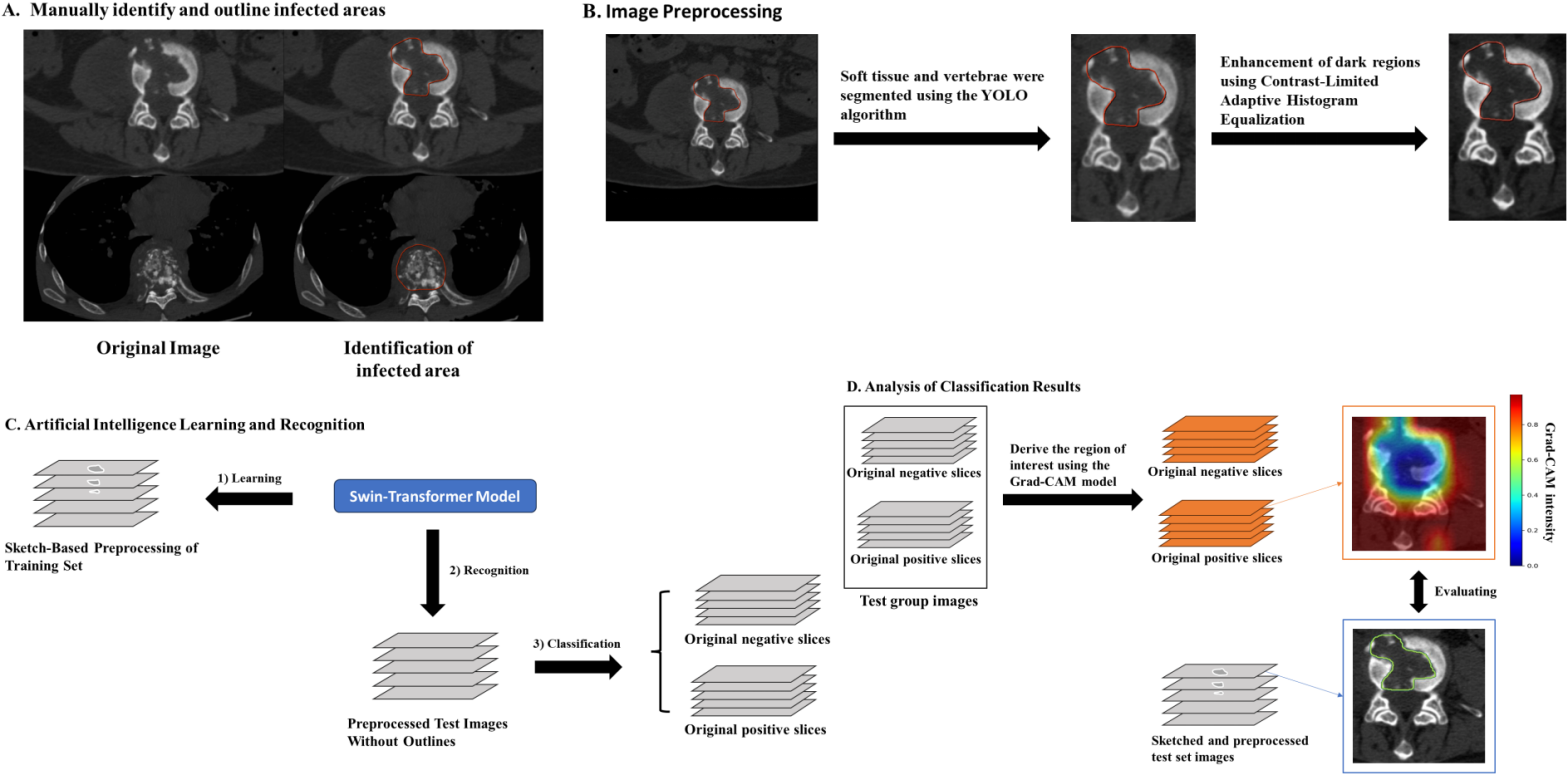
Workflow of the deep learning model development.

All selected images were segmented for the vertebrae (intervertebral disc) and surrounding tissues using the YOLO algorithm. Subsequently, the darker regions of the images were enhanced to varying degrees by employing the Contrast-Limited Adaptive Histogram Equalization model. The Swin-Transformer model was then utilized to identify the spinal infections. Finally, the Gradient-weighted Class Activation Mapping (Grad-CAM) derived the region of interest to ensure the results of the Swin-Transformer model are correct. as shown in Fig. 2. In addition, to improve sample diversity and model robustness, data augmentation was employed exclusively during the training phase. The augmentation strategies comprised: (i) geometric transformations (random affine, cropping, and flipping); (ii) intensity/contrast perturbations (*e.g.*, contrast enhancement); and (iii) mild imaging degradation simulations (*e.g.*, blurring/sharpening). These operations were applied on-the-fly to increase the effective sample space and reduce the model’s reliance on specific imaging conditions. No stochastic augmentations were introduced during validation or external testing, ensuring deterministic and reproducible evaluation. To address the severe class imbalance (positive: negative slice ratio ≈ 1:9), we applied random oversampling of positive slices exclusively to the training set and optimized the model using binary cross-entropy loss with L2 weight decay (*λ* = 5 × 10^−4^).

After model training, we calculated a prediction score for each suspected lesion area, which was then visualized as bounding boxes and pixel-level heatmaps overlaid on the original images (Fig. 2). These scores were subsequently dichotomized using a threshold of 0.5 to classify each area as either negative (score <0.5) or positive for spinal infection (score ≥ 0.5).

### CT interpretation by radiologists

Two radiologists with distinct levels of clinical experience, neither of whom participated in the image segmentation process and both blinded to all clinical, pathological, and microbiological information, independently evaluated the CT images in the internal and external validation sets. They performed classification at two complementary levels: slice level and patient level. For slice-level assessment, each radiologist recorded the presence, location, and morphological type of any suspected infectious findings (*e.g.*, vertebral endplate erosion, disc involvement, paravertebral soft tissue abnormalities, or epidural abscess). For patient-level classification, a patient was considered positive for spinal infection if at least one axial slice exhibited any of the above findings; otherwise, the patient was classified as negative. Any discrepancies between the two readers were resolved through consensus discussion.

### Statistical analysis

Statistical analyses were performed using R software (version 4.2.2). Continuous variables are expressed as mean ± standard deviation, and categorical variables as counts and percentages. The diagnostic performance of the deep learning (DL) model and radiologists was evaluated on a per-axial CT slice basis using the area under the receiver operating characteristic curve (AUC), area under the precision-recall curve (AUPRC), sensitivity, specificity, accuracy, positive predictive value (PPV), negative predictive value (NPV), and F1 score, with 95% confidence intervals (CIs) computed *via* the DeLong method for AUC, bootstrap resampling (1,000 iterations) for AUPRC, and the Clopper-Pearson exact method for binary metrics. Comparisons of AUC between the DL model and each radiologist, as well as between radiologists with *versus* without DL assistance, were conducted using DeLong’s test for correlated ROC curves; AUPRC differences were assessed *via* bootstrap-based testing. For sensitivity, specificity, accuracy, NPV, and F1 score, McNemar’s test was applied to paired binary predictions, while PPV comparisons were performed using the chi-square test. Reading times (seconds) were compared using the independent-samples *t*-test for DL *versus* radiologists and the paired-samples *t*-test for the same radiologist with and without AI assistance, after verifying normality with the Shapiro-Wilk test. Subgroup analyses stratified by sex, age, infection level, CT protocol, pathogen, and spinal epidural abscess presence were performed, and AUC differences between subgroups were evaluated using DeLong’s test. All tests were two-tailed, and *P* < 0.05 was considered statistically significant.

## Results

### Patient characteristic

The patient selection process is illustrated in Fig. 1. Initially, 213 patients from the primary center were screened, and 86 were excluded according to the predefined criteria, resulting in 127 eligible patients. These patients were then randomly divided into a training set (*n* = 90) and an internal validation set (*n* = 37) at an approximate ratio of 7:3. Additionally, 30 patients from an external center were included as an external validation cohort. The baseline characteristics of all enrolled patients are summarized in Table 1.

**Table 1:** Patient characteristics of the training and validation cohorts (*n* = 157).

Variables	Training cohort (*n* = 90)	Internal validation cohort (*n* = 37)	External validation cohort (*n* = 30)
Age (years)	59.39 ± 13.54	63.14 ± 13.41	63.33 ± 9.72
Sex			
Male (*n* = 93)	54 (60.00%)	21 (56.76%)	18 (60.00%)
Female (*n* = 64)	36 (40.00%)	16 (43.24%)	12 (40.00%)
Body mass index (kg/m^2^)	22.70 ± 4.16	21.25 ± 3.67	22.76 ± 4.41
CT Scan Type			
Thoracoabdominal (*n* = 46)	24 (26.67%)	9 (24.32%)	13 (43.33%)
Dedicated Spine (*n* = 111)	66 (73.33%)	28 (75.68%)	17 (57.67%)
Pathogen			
Mycobacterium tuberculosis (*n* = 30)	20 (22.22%)	5 (13.51%)	5 (16.67%)
Non-tuberculous infections (*n* = 127)	70 (77.78%)	32 (86.49%)	25 (83.33%)
Location of Infection			
Cervical/Thoracic (*n* = 62)	37 (41.11%)	14 (37.84%)	10 (33.33%)
Lumbar/Sacral (*n* = 95)	53 (58.89%)	23 (62.16%)	20 (66.67%)
Complication			
Spinal epidural abscess			
With (*n* = 69)	40 (44.44%)	14 (37.84%)	15 (50.00%)
Without (*n* = 88)	50 (55.56%)	23 (62.16%)	15 (50.00%)
Psoas major muscle infection			
With (*n* = 14)	6 (6.67%)	2 (8.11%)	6 (20.00%)
Without (*n* = 143)	84 (93.33%)	35 (91.89%)	24 (80.00%)

**Notes.**

Data are presented as mean ± standard deviation or number of patients (%).

Within the entire primary cohort (*n* = 127), 30 patients (23.6%) had spondylodiscitis caused by *Mycobacterium tuberculosis*, while 97 patients (76.4%) had non-tuberculous infections. Regarding the anatomical level of infection, 61 patients (48.0%) presented with cervical or thoracic involvement, and 66 patients (52.0%) had lumbar or sacral involvement. In the internal validation set (*n* = 37), five patients (13.5%) had tuberculous spondylodiscitis and 32 (86.5%) had non-tuberculous infections; 14 patients (37.8%) had cervical/thoracic infections and 23 (62.2%) had lumbar/sacral infections. The external validation cohort (*n* = 30) consisted of 5 patients (16.7%) with tuberculous and 25 (83.3%) with non-tuberculous infections; 10 patients (33.3%) had cervical/thoracic involvement and 20 (66.7%) had lumbar/sacral involvement (Table 1).

### Multiple comparisons of deep learning methods

Since the thoracic-abdominal or spinal transect slices contain a variety of information (including intestines, lungs, heart, *etc*.), the vertebrae only account for a small portion of the original image. Therefore, we first used the yolo algorithm to accomplish the accurate segmentation of vertebrae (see Fig. 2B), with a segmentation accuracy of 99.8% (18,668/18,705). Thereafter we found that there were differences in light and darkness in the resulting CT slices due to differences in the machines, so we used the clahe algorithm to adaptively enhance the dark parts of the slices to achieve consistent lightness and darkness (Fig. 2C). Thereafter we used Efficientnet-b0, Resnet, Swin-Transformer, ViT, Pvt, Deit, Densenet for identification of spinal infections on transected slices. In the training group, Swin-transformer efficacy was the highest. Therefore, we used Swin-Transformer for spinal infection lesion identification. To verify whether it was the identification of the vertebral infection site that led to the final result, we used Grad-CAM to analyze the images to see if the focus of attention was correctly focused on the lesion area. Swin-Transformer (AUC = 97.4%, ACC = 91.1%, SPE = 90.1%, SEN = 93.9%) had better diagnostic results than other depth models such as Efficientnet-b0 (AUC = 95.2%, ACC = 95.7%, SPE = 98.4%, SEN = 58.6%)

### Diagnostic performance of the deep learning-based Approach

**Figure 3:**
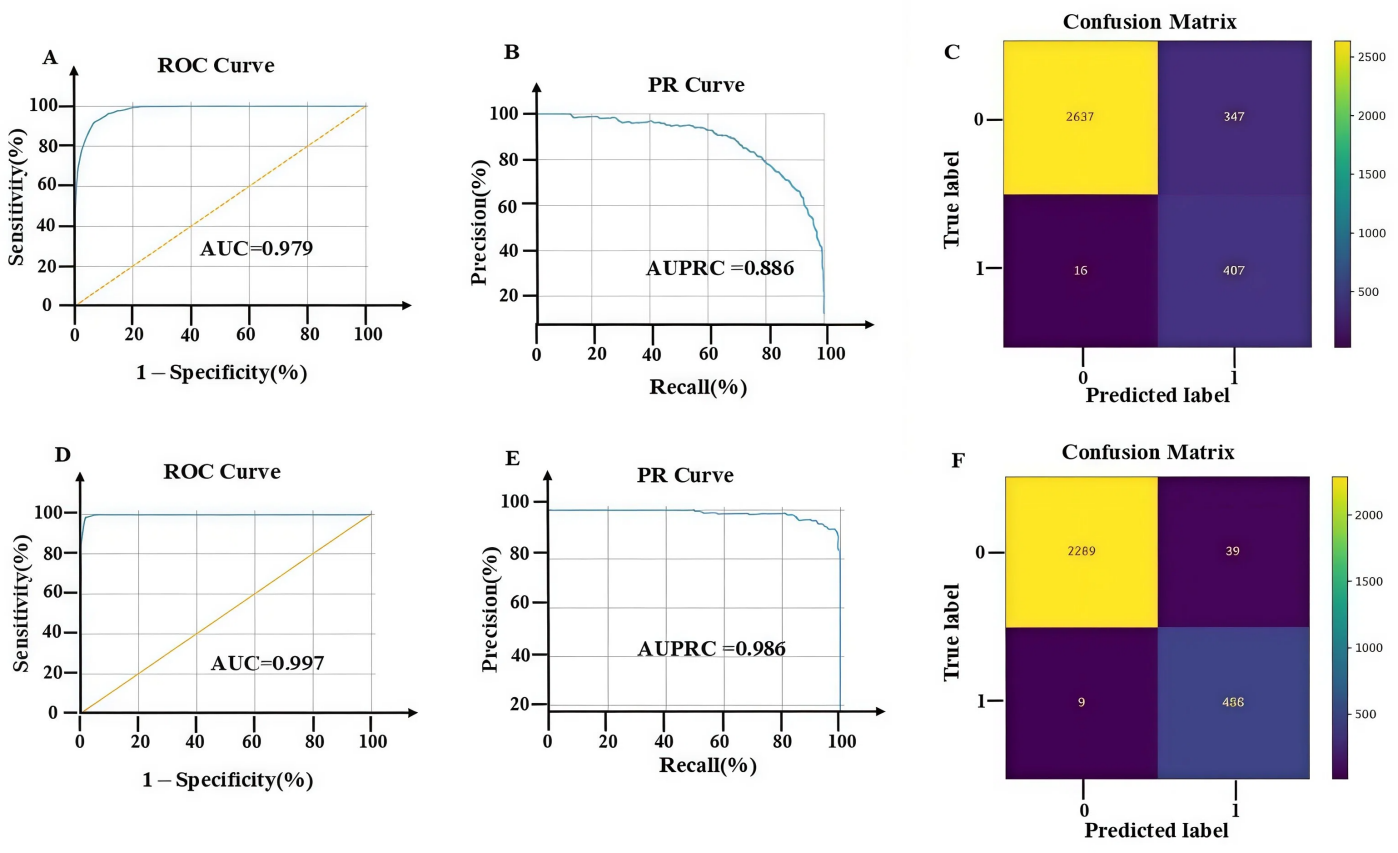
Diagnostic performance of the deep learning model and radiologists in the internal and external validation cohorts. (A–C) Internal validation cohort (*n* = 37). (D–F) External validation cohort (*n* = 30). (A, D) ROC curves. The solid lines represent the deep learning model (blue). The dashed diagonal line indicates the line of no discrimination. AUC values are provided in the legend. (B, E) PR. The solid lines represent the deep learning model (blue). AUPRC values are shown in the legend. (C, F) Slice-level confusion matrices for the deep learning model. Rows correspond to true labels and columns to predicted labels (0: negative, 1: positive). Each cell displays the raw count of true positives (TP), false positives (FP), false negatives (FN), and true negatives (TN). Color intensity is scaled to the maximum count in each matrix for visual clarity. Abbreviations: AUC, area under the receiver operating characteristic curve; AUPRC, area under the precision–recall curve; CI, confidence interval; DL, deep learning; ROC, receiver operating characteristic; PR, precision–recall.

The diagnostic performance of the deep learning -based approach in the test group is shown in Fig. 3. All performance metrics (AUC, sensitivity, specificity, accuracy) are reported per axial CT slice.

The diagnostic performance of the DL model and two radiologists is summarized in Table 2 (internal) and Table 3 (external).

**Table 2:** Diagnostic performance of the deep learning model and radiologists on a per-axial CT slice basis in the internal validation cohort.

	DL	Radiologist 1	Radiologist 2
AUC (95% CI)	0.979 (0.971–0.986)	0.704 (0.594–0.827)	0.584 (0.536–0.643)
*P* value		<0.001	<0.001
AUPRC (95% CI)	0.886 (0.833–0.928)	0.410 (0.303–0.570)	0.286 (0.188–0.406)
*P* value		<0.001	<0.001
Precision (PPV) (95% CI)	0.540 (0.475–0.618)	0.682 (0.522–0.900)	0.750 (0.454–1.000)
*P* value		>0.05	>0.05
NPV (95% CI)	0.994 (0.991–0.997)	0.886 (0.794–0.954)	0.843 (0.775–0.895)
*P* value		>0.05	<0.05
Sensitivity (95% CI)	0.962 (0.938–0.981)	0.455 (0.227–0.714)	0.182 (0.077–0.308)
*P* value		<0.001	<0.001
Specificity (95% CI)	0.905 (0.895–0.914)	0.896 (0.872–0.916)	0.899 (0.875–0.918)
*P* value		>0.05	>0.05
Accuracy (95% CI)	0.894 (0.867–0.919)	0.861 (0.783–0.922)	0.839 (0.772–0.889)
*P* value		>0.05	>0.05
F1 (95% CI)	0.692 (0.635–0.753)	0.546 (0.333–0.708)	0.293 (0.143–0.444)
*P* value		<0.001	<0.001

**Notes.**

Data are presented as point estimates with 95% CIs in parentheses.

*P* value for diagnostic performance difference between DL model and radiologists.

Abbreviations DL Deep Learning CI Confidence Interval AUC Area Under the ROC Curve AUPRC Area Under the Precision-Recall Curve PPV Positive Predictive Value NPV Negative Predictive Value

**Table 3:** Diagnostic performance of the deep learning model and radiologists on a per-axial CT slice basis in the external validation cohort.

	DL	Radiologist 1	Radiologist 2
AUC (95% CI)	0.989(0.975–0.998)	0.663(0.593–0.753)	0.648(0.579–0.715)
*P* value		<0.001	<0.001
AUPRC (95% CI)	0.972(0.956–0.988)	0.317(0.235–0.426)	0.331(0.220–0.455)
*P* value		<0.001	<0.001
Precision (PPV)(95% CI)	0.926(0.888–0.957)	0.553(0.418–0.686)	0.667(0.482–0.818)
*P* value		<0.001	<0.001
NPV(95% CI)	0.996(0.993–0.999)	0.884(0.827–0.931)	0.877(0.830–0.917)
*P* value		>0.05	<0.05
Sensitivity (95% CI)	0.982(0.967–0.993)	0.388(0.250–0.563)	0.328(0.195–0.464)
*P* value		<0.001	<0.001
Specificity (95% CI)	0.983(0.974–0.991)	0.937(0.912–0.960)	0.967(0.948–0.984)
*P* value		>0.05	>0.05
Accuracy (95% CI)	0.983(0.976–0.989)	0.845(0.795–0.890)	0.860(0.813–0.900)
*P* value		<0.05	<0.05
F1 (95% CI)	0.953(0.931–0.970)	0.456(0.323–0.591)	0.440(0.280–0.574)
*P* value		<0.001	<0.001

**Notes.**

Data are presented as point estimates with 95% CIs in parentheses.

*P* value for diagnostic performance difference between DL model and radiologists.

Abbreviations DL Deep Learning CI Confidence Interval AUC Area Under the ROC Curve AUPRC Area Under the Precision-Recall Curve PPV Positive Predictive Value NPV Negative Predictive Value

In the internal validation set, the DL model achieved an AUC of 0.979, AUPRC of 0.886, sensitivity of 96.2%, specificity of 90.5%, accuracy of 89.4%, PPV of 54.0%, NPV of 99.4%, and F1 score of 69.2%. Both radiologists showed significantly lower AUC, AUPRC, sensitivity, and F1 score (all *P* < 0.001). NPV was comparable for Radiologist 1 (*P* > 0.05) but significantly lower for Radiologist 2 (*P* < 0.05). PPV, Specificity and accuracy did not differ significantly (both *P* > 0.05).

In the external validation set, the DL model demonstrated excellent and well- generalized performance: AUC 0.989, AUPRC 0.972, sensitivity 98.2%, specificity 98.3%, accuracy 98.3%, PPV 92.6%, NPV 99.6%, and F1 score 95.3%. Both radiologists were significantly inferior in AUC, AUPRC, sensitivity, accuracy, and F1 score (all *P* < 0.001), while specificity was comparable (both *P* > 0.05). The DL model also achieved significantly higher PPV than Radiologist 1 (55.3%, *P* < 0.001) and similar PPV to Radiologist 2 (66.7%, *P* > 0.05). Its NPV was comparable to Radiologist 1 (88.4%, *P* > 0.05) but significantly higher than that of Radiologist 2 (87.7%, *P* < 0.05).

These findings confirm the strong generalizability of the DL model, with consistently superior diagnostic performance across independent cohorts.

### The deep learning -based approach helps enhance radiologists’ diagnoses

Deep learning (DL)-based assistance significantly improved radiologists’ diagnostic performance across multiple metrics.

In the internal test set, Radiologist 1 demonstrated substantial gains, with AUC increasing from 0.704 to 0.924, AUPRC from 0.410 to 0.682, sensitivity from 45.5% to 91.8%, accuracy from 86.1% to 92.8%, and F1 score from 54.6% to 81.2% (all *P* < 0.001). NPV improved from 88.6% to 98.2% (*P* < 0.05).

**Figure 4:**
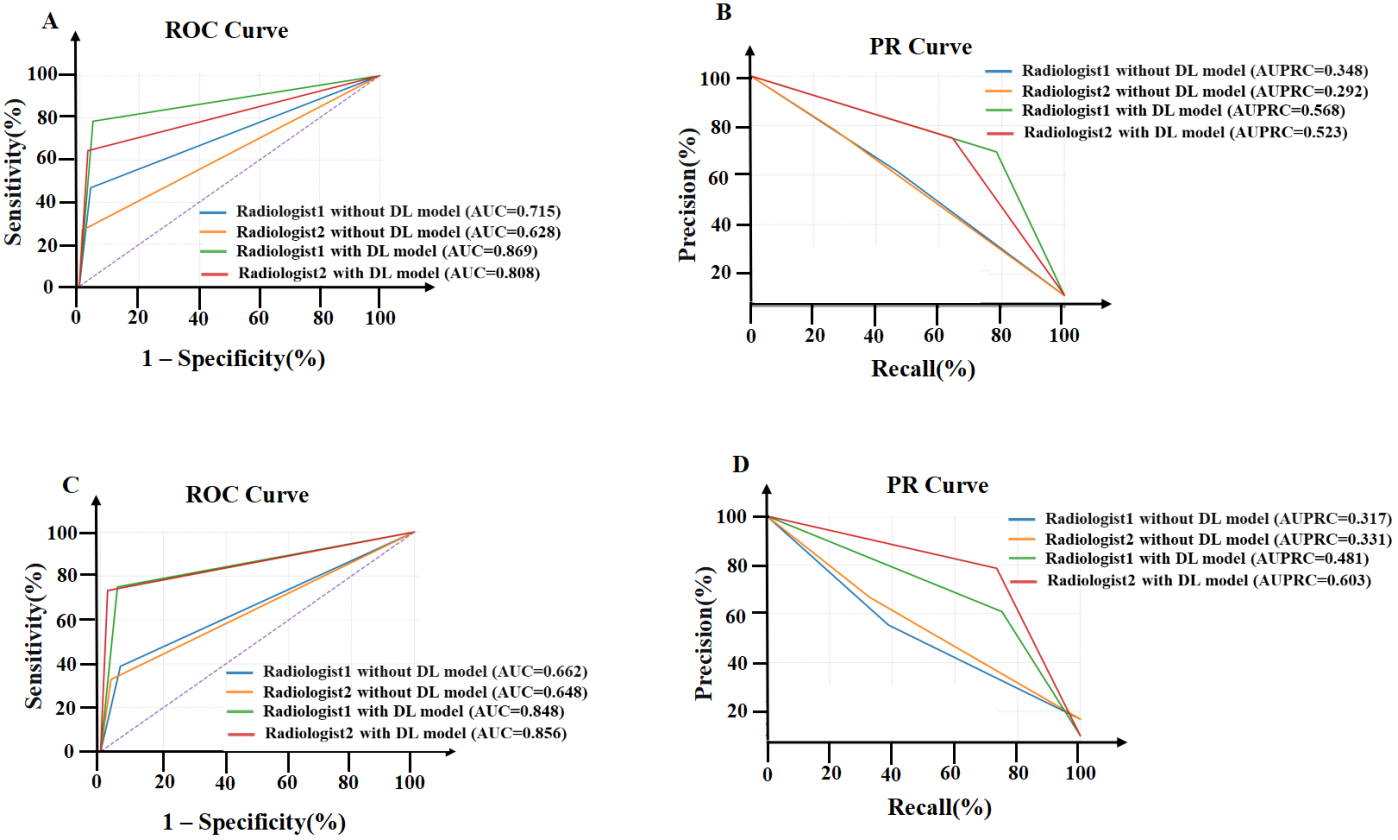
Diagnostic performance of radiologists with and without deep learning assistance. (A, B) Internal validation cohort (*n* = 37). (C, D) External validation cohort (*n* = 30). (A, C) ROC curves comparing Radiologist 1 and Radiologist 2 with and without DL assistance. The diagonal dotted line represents the line of no discrimination.AUC valuesare provided in the figure legend. (B, D) PR curves comparing Radiologist 1 and Radiologist 2 with and without DL assistance. AUPRC values are shown in the figure legend. Abbreviations: AUC, area under the receiver operating characteristic curve; AUPRC, area under the precision–recall curve; DL, deep learning; ROC, receiver operating characteristic; PR, precision–recall.

Radiologist 2 also improved markedly: AUC rose from 0.584 to 0.765, AUPRC from 0.286 to 0.463, sensitivity from 18.2% to 59.0%, accuracy from 83.9% to 88.1%, and F1 score from 29.3% to 62.6% (*P* < 0.05). NPV increased from 84.3% to 91.8% (*P* < 0.05) (see Table 4, Fig. 4).

**Table 4:** Diagnostic performance of radiologists without and with DL-based assistance on a per-axial CT slice basis in the internal validation cohort.

	Radiologist 1		Radiologist 2	
	With DL model	Without DL model	With DL model	Without DL model
AUC (95% CI)	0.924(0.862–0.973)	0.704(0.594–0.827)	0.765(0.607–0.892)	0.584(0.536–0.643)
*P* value	<0.001		<0.001	
AUPRC (95% CI)	0.682(0.412–0.910)	0.410(0.303–0.570)	0.463(0.208–0.776)	0.286(0.188–0.406)
*P* value	<0.001		<0.001	
Precision (PPV)(95% CI)	0.727(0.451–0.961)	0.682(0.522–0.900)	0.750(0.455–1.000)	0.667(0.313–1.000)
*P* value	>0.05		>0.05	
NPV(95% CI)	0.982(0.965–1.000)	0.886(0.794–0.954)	0.918(0.850–0.974)	0.843(0.775–0.895)
*P* value	<0.05		<0.05	
Sensitivity (95% CI)	0.918(0.825–1.000)	0.455(0.227–0.714)	0.590(0.293–0.857)	0.182(0.077–0.308)
*P* value	<0.001		<0.001	
Specificity (95% CI)	0.930(0.843–0.990)	0.952(0.908–0.987)	0.940(0.842–1.000)	0.986(0.957–1.000)
*P* value	>0.05		>0.05	
Accuracy (95% CI)	0.928(0.853–0.981)	0.861(0.783–0.922)	0.881(0.792–0.953)	0.839(0.772–0.889)
*P* value	>0.05		>0.05	
F1 (95% CI)	0.812(0.594–0.948)	0.546(0.333–0.708)	0.626(0.333–0.849)	0.293(0.143–0.444)
*P* value	<0.001		<0.05	

**Notes.**

Data are presented as point estimates with 95% CIs in parentheses.

*P* value for diagnostic performance difference between radiologists with DL model and without DL model.

Abbreviations DL Deep Learning CI Confidence Interval AUC Area Under the ROC Curve AUPRC Area Under the Precision-Recall Curve PPV Positive Predictive Value NPV Negative Predictive Value

The benefit was even more pronounced in the external validation cohort. With DL assistance, Radiologist 1 achieved an AUC increase from 0.663 to 0.848, AUPRC from 0.317 to 0.481, sensitivity from 38.8% to 75.0%, accuracy from 84.5% to 92.7%, and F1 score from 45.6% to 67.2% (all *P* < 0.001). PPV rose from 55.3% to 60.8% (*P* < 0.01), and NPV rose from 88.4% to 97.2% (*P* < 0.05).

Radiologist 2 also showed substantial improvements: AUC increased from 0.648 to 0.856, AUPRC from 0.331 to 0.603, sensitivity from 32.8% to 73.3%, accuracy from 86.0% to 95.3%, and F1 score from 44.0% to 75.9% (*P* < 0.001 for AUC, AUPRC, sensitivity; *P* < 0.01 for accuracy and F1). PPV increased from 66.7% to 78.6% (*P* < 0.01), and NPV increased from 87.7% to 97.1% (*P* < 0.05). (see Table 5, Fig. 4).

**Table 5:** Diagnostic performance of radiologists without and with DL-based assistance on a per-axial CT slice basis in the external validation cohort.

	Radiologist 1		Radiologist 2	
	With DL model	Without DL model	With DL model	Without DL model
AUC (95% CI)	0.848(0.785–0.903)	0.663(0.593–0.753)	0.856(0.788–0.913)	0.648(0.579–0.715)
*P* value	<0.001		<0.001	
AUPRC (95% CI)	0.481(0.322–0.641)	0.317(0.235–0.426)	0.603(0.427–0.743)	0.331(0.220–0.455)
*P* value	<0.001		<0.001	
Precision (PPV)(95% CI)	0.608(0.434–0.788)	0.553(0.418–0.686)	0.786(0.610–0.915)	0.667(0.482–0.818)
*P* value	<0.01		<0.01	
NPV(95% CI)	0.972(0.955–0.986)	0.884(0.827–0.931)	0.971(0.952–0.985)	0.877(0.830–0.917)
*P* value	<0.05		<0.05	
Sensitivity (95% CI)	0.750(0.622–0.857)	0.388(0.250–0.563)	0.733(0.597–0.851)	0.328(0.195–0.464)
*P* value	<0.001		<0.001	
Specificity (95% CI)	0.946(0.913–0.975)	0.937(0.912–0.960)	0.978(0.965–0.991)	0.967(0.948–0.984)
*P* value	>0.05		>0.05	
Accuracy (95% CI)	0.927(0.897–0.954)	0.845(0.795–0.890)	0.953(0.939–0.969)	0.860(0.813–0.900)
*P* value	<0.05		<0.01	
F1 (95% CI)	0.672(0.531–0.786)	0.456(0.323–0.591)	0.759(0.630–0.848)	0.440(0.280–0.574)
*P* value	<0.05		<0.01	

**Notes.**

Data are presented as point estimates with 95% CIs in parentheses.

*P* value for diagnostic performance difference between radiologists with DL model and without DL model.

Abbreviations DL Deep Learning CI Confidence Interval AUC Area Under the ROC Curve AUPRC Area Under the Precision-Recall Curve PPV Positive Predictive Value NPV Negative Predictive Value

These findings confirm that DL-based assistance consistently and substantially enhances radiologists’ diagnostic accuracy, with robust generalizability evidenced in an independent external validation setting.

In addition to slice- level evaluation, we assessed patient-level diagnostic performance using an expanded cohort comprising 37 infected patients and 127 independent non-infected controls. The DL model achieved 100% sensitivity and 99.2% specificity, with performance equivalent to both radiologists (all *P* > 0.05; Table S2, Fig. 3). Detailed patient-level analysis is provided in the Supplementary Materials.

### T**hedeep learning-based approach reduces diagnostic time**

Tables 6 and 7 summarize the image interpretation times of the deep learning model and radiologists, with and without AI assistance, in both the internal and external validation cohorts.

**Table 6:** Comparison of image interpretation times between the deep learning model and radiologists on a per-axial CT slice basis in the internal validation cohort.

	Without DL model		With DL model	
	Time(s)	*P* Value^*^	Time(s)	*P* Value^#^
DL model	0.019 ± 0.006			
Radiologist1	0.650 ± 0.169	<0.001	0.606 ± 0.229	<0.001
Radiologist2	0.567 ± 0.160	<0.001	0.538 ± 0.253	<0.001

**Notes.**

Abbreviations DL Deep Learning CT Computed tomography

* *P* value for reading time difference between DL model and radiologists.

# *P* value for reading time difference between with and without DL model.

**Table 7:** Comparison of image interpretation times between the deep learning model and radiologists on a per-axial CT slice basis in the external validation cohort.

	Without DL model		With DL model	
	Time(s)	*P* Value^*^	Time(s)	*P* Value^#^
DL model	0.019 ± 0.005			
Radiologist1	1.092 ± 0.636	<0.001	0.866 ± 0.393	<0.001
Radiologist2	1.077 ± 0.178	<0.001	0.784 ± 0.474	<0.001

**Notes.**

DL Deep Learning

* *P* value for reading time difference between DL model and radiologists.

# *P* value for reading time difference between with and without DL model.

In the internal cohort, the DL model completed diagnosis in 0.019 ± 0.006 s, significantly faster than both radiologists (0.650 ± 0.169 s and 0.567 ± 0.160 s; both *P* < 0.001). With DL assistance, reading times of Radiologist 1 and Radiologist 2 decreased to 0.606 ± 0.229 s and 0.538 ± 0.253 s, respectively (both *P* < 0.001 *vs.* without assistance).

This efficiency gain was well preserved in the external validation cohort, demonstrating strong generalizability. The DL model maintained a rapid inference time of 0.019 ± 0.005 s. Without assistance, radiologists required 1.092 ± 0.636 s (radiologist 1) and 1.077 ± 0.178 s (radiologist 2). With DL aid, their reading times significantly dropped to 0.866 ±  0.393 s and 0.784 ± 0.474 s, respectively (both *P* < 0.001). These results confirm that DL-based assistance consistently and substantially reduces radiologists’ diagnostic time across independent cohorts.

### Subgroup snalysis by CT scan type

**Figure 5:**
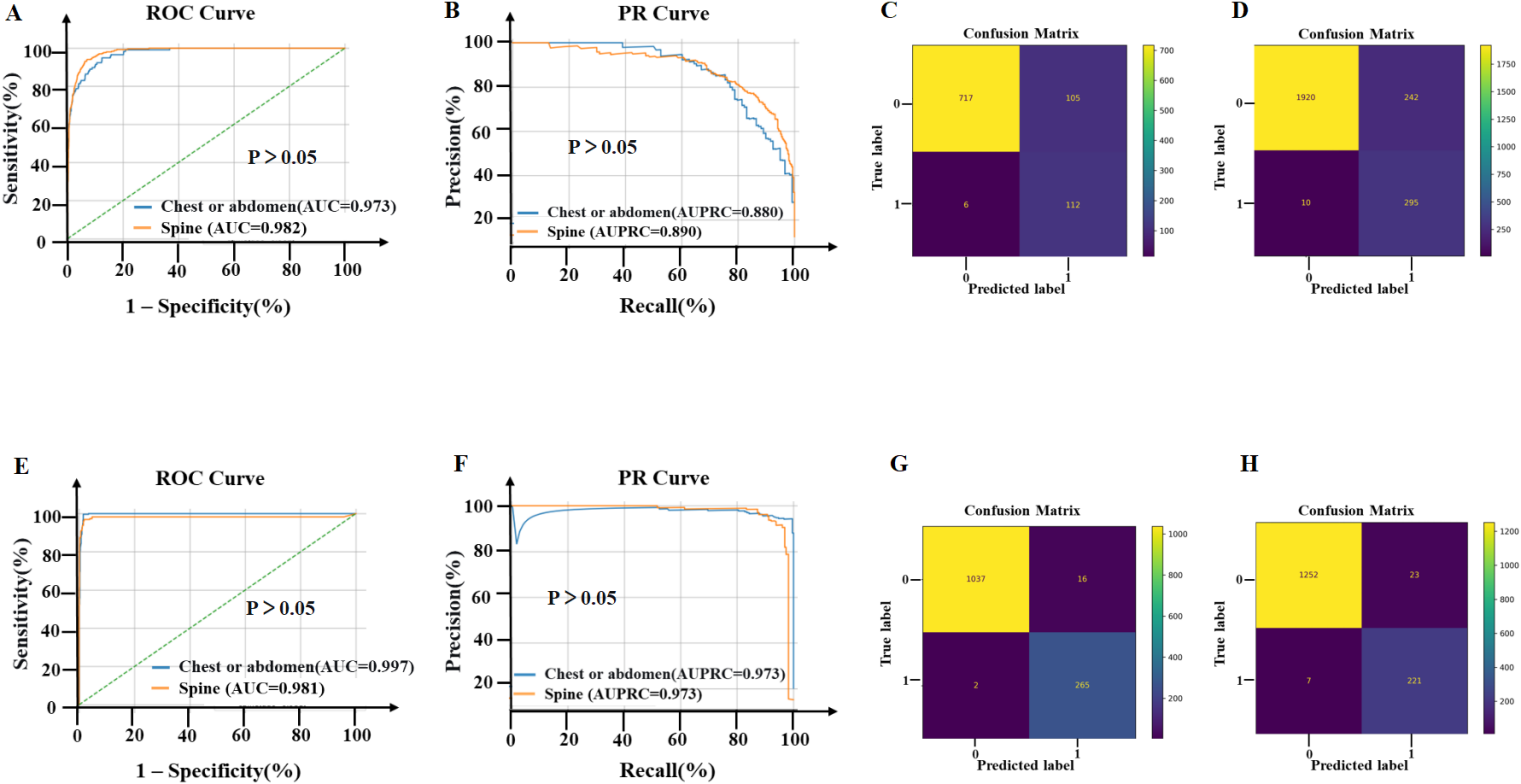
Subgroup analysis of DL model diagnostic performance stratified by CT scan type (dedicated spine CT *vs.* thoracoabdominal CT). (A–D) Internal validation cohort. (A) ROC curves: dedicated spine CT (orange, AUC = 0.982), thoracoabdominal CT (blue, AUC = 0.973); *P* > 0.05. (B) PR curves: dedicated spine CT (orange, AUPRC = 0.890), thoracoabdominal CT (blue, AUPRC = 0.880); *P* > 0.05. (C) Slice-level confusion matrix for thoracoabdominal CT. (D) Slice-level confusion matrix for dedicated spine CT. (E–H) External validation cohort. (E) ROC curves: dedicated spine CT (orange, AUC = 0.981), thoracoabdominal CT (blue, AUC = 0.997); *P* > 0.05. (F) PR curves: dedicated spine CT (orange, AUPRC = 0.973), thoracoabdominal CT (blue, AUPRC = 0.973); *P* > 0.05. (G) Slice-level confusion matrix for thoracoabdominal CT. (H) Slice-level confusion matrix for dedicated spine CT. Abbreviations: DL, deep learning; AUC, area under the receiver operating characteristic curve; AUPRC, area under the precision-recall curve; CT, computed tomography; ROC, receiver operating characteristic; PR, precision-recall. *P* values for AUC comparisons were derived from DeLong’s test for paired ROC curves; *P* values for AUPRC comparisons were derived from bootstrap-based hypothesis testing.

We further evaluated the diagnostic performance of the deep learning model stratified by CT acquisition protocol in both the internal validation and external validation cohorts (Tables 8 and 9, Fig. 5).

**Table 8:** Subgroup analysis of DL model diagnostic performance stratified by CT scan type in the internal validation cohort.

CT Scan Type	Dedicated Spine	Thoracoabdominal
AUC (95% CI)	0.982(0.972–0.990)	0.973(0.960–0.985)
*P* value		>0.05
AUPRC (95% CI)	0.890(0.819–0.941)	0.880(0.790–0.940)
*P* value		>0.05
Precision (95% CI)	0.549(0.462–0.643)	0.516(0.427–0.678)
*P* value		>0.05
NPV(95% CI)	0.995(0.991–0.998)	0.992(0.986–0.997)
*P* value		>0.05
Sensitivity (95% CI)	0.967(0.945–0.987)	0.949(0.883–0.987)
*P* value		>0.05
Specificity (95% CI)	0.888(0.851–0.918)	0.872(0.805–0.937)
*P* value		>0.05
Accuracy (95% CI)	0.898(0.866–0.925)	0.882(0.831–0.936)
*P* value		>0.05
F1 (95% CI)	0.701(0.625–0.773)	0.669(0.585–0.787)
*P* value		>0.05

**Notes.**

Data are presented as point estimates with 95% CIs in parentheses.

*P* values were derived from the comparison of diagnostic performance between dedicated spine and thoracoabdominal CT acquisitions.

Abbreviations DL Deep Learning CI Confidence Interval AUC Area Under the ROC Curve AUPRC Area Under the Precision-Recall Curve PPV Positive Predictive Value NPV Negative Predictive Value

**Table 9:** Subgroup analysis of DL model diagnostic performance stratified by CT scan type in the external validation cohort.

CT Scan type	Dedicated spine	Thoracoabdominal
AUC (95% CI)	0.981(0.954–0.999)	0.997(0.994–0.999)
*P* value		>0.05
AUPRC (95% CI)	0.973(0.942–0.994)	0.973(0.960–0.992)
*P* value		>0.05
Precision (95% CI)	0.906(0.840–0.960)	0.943(0.909–0.971)
*P* value		>0.05
NPV(95% CI)	0.994(0.990–0.998)	0.998(0.996–1.000)
*P* value		>0.05
Sensitivity (95% CI)	0.969(0.942–0.992)	0.993(0.985–1.000)
*P* value		>0.05
Specificity (95% CI)	0.982(0.969–0.993)	0.985(0.971–0.993)
*P* value		>0.05
Accuracy (95% CI)	0.980(0.969–0.989)	0.986(0.977–0.993)
*P* value		>0.05
F1 (95% CI)	0.936(0.899–0.967)	0.967(0.951–0.980)
*P* value		>0.05

**Notes.**

Data are presented as percentage with 95% confidence intervals in parentheses.

*P* values were derived from the comparison of diagnostic performance between dedicated spine and thoracoabdominal CT acquisitions.

Abbreviations DL Deep Learning CI Confidence Interval AUC Area Under the ROC Curve AUPRC Area Under the Precision-Recall Curve PPV Positive Predictive Value NPV Negative Predictive Value

In the internal validation cohort, the model demonstrated comparable performance between dedicated spine CT and thoracoabdominal CT across all metrics: AUC (0.982 *vs.* 0.973), AUPRC (0.890 *vs.* 0.880), sensitivity (96.7% *vs.* 94.9%), specificity (88.8% *vs.* 87.2%), and accuracy (89.8% *vs.* 88.2%), with no statistically significant differences observed (all *P* > 0.05).

Similarly, in the external validation cohort, the model maintained high and consistent performance across both CT types. For dedicated spine CT *versus* thoracoabdominal CT, the model achieved AUC of 0.981 *vs.* 0.997, sensitivity of 96.9% *vs.* 99.3%, specificity of 98.2% *vs.* 98.5%, and accuracy of 98.0% *vs.* 98.6%, respectively. All comparisons between the two subgroups were non-significant (all *P* > 0.05).

These findings indicate that the deep learning model maintains robust diagnostic accuracy across different CT acquisition protocols, supporting its potential applicability to routine thoracoabdominal CT scans where spinal infections are often incidentally detected.

### Factors affect the diagnostic efficiency of deep learning

We further investigated whether various clinical and imaging factors influenced the diagnostic performance of the deep learning model, including infection level, CT protocol, presence of spinal epidural abscess (SEA), pathogen type, sex, and age. Among these, the presence of SEA and the type of pathogen were identified as significant influencing factors (both *P* < 0.001; Table 10).

**Table 10:** Factors influencing the diagnostic performance of the deep learning model.

	AUC (95% CI)	*P*
Sex		
Male	0.977(0.970–0.983)	
Female	0.984(0.977–0.991)	0.140
Age (years)		
<60	0.979 (0.974–0.985)	
>60	0.980(0.969–0.991)	0.938
Location of Infection		
Cervical/Thoracic	0.983(0.973–0.992)	
Lumbar/Sacral	0.978(0.972–0.984)	0.433
CT Scan Type		
Dedicated Spine	0.978(0.973–0.983)	
Thoracoabdominal	0.981(0.970–0.992)	0.940
Pathogen		
*Mycobacterium tuberculosis*	0.979(0.974–0.984)	
Non-tuberculous infections	0.997(0.992–0.999)	<0.001
SEA		
With SEA	0.950(0.941–0.959)	
Without SEA	0.783(0.611–0.954)	<0.001

**Notes.**

Abbreviations AUC area under the receiver operating characteristic curve CI confidence interval SEA spinal epidural abscess

*P* values were derived from the comparison of AUCs between subgroups within each factor using the DeLong test for two correlated ROC curves.

### Factors affect the diagnostic efficiency of deep learning

We further investigated whether various clinical and imaging factors influenced the diagnostic performance of the deep learning model, including infection level, CT protocol, presence of spinal epidural abscess (SEA), pathogen type, sex, and age. Among these, the presence of SEA and the type of pathogen were identified as significant influencing factors (both *P* < 0.001; Table 10).

### Interpretability of the model

To enhance the transparency and clinical trustworthiness of the deep learning model, Gradient-weighted Class Activation Mapping (Grad-CAM) was employed to visualize the regions of interest driving the model’s predictions. As shown in Fig. 2, the Grad-CAM heatmaps consistently highlighted areas corresponding to vertebral osteomyelitis (indicated in blue), such as vertebral endplate erosion, paravertebral soft tissue involvement, and epidural abscess formation. These attention patterns showed strong alignment with key diagnostic features used by radiologists, including loss of vertebral height, bone destruction, and adjacent soft tissue inflammation. The spatial concordance between model attention and radiological landmarks underscores the model’s ability to learn clinically relevant representations and supports its validity as a diagnostic tool for spinal infections on non-contrast CT.

## Discussion

Early diagnosis is critical to prevent further complications of spinal infections; however, spinal infections are often asymptomatic or have nonspecific symptoms that make identification clinically difficult (Babic, Simpfendorfer & Berbari, 2019). Although MRI is the preferred imaging examination for diagnosing spinal infections (Berbari et al., 2015), non-contrast CT is more readily available for initial screening and emergency patients. For patients with concomitant thoracoabdominal diseases, thoracoabdominal CT transects are an important imaging tool for early screening of spinal infections. However, spinal infections incidentally detected on routine thoracoabdominal CT are often underreported due to the lack of sagittal spinal reconstruction and the limited knowledge of the spine among thoracoabdominal radiologists. In addition, identification of spinal infections by non-contrast CT images is challenging and requires experienced radiologists and additional time for analysis (Cardoso et al., 2020; Salaffi et al., 2021). Even experienced radiologists specializing in bone and soft tissues still have a certain rate of underdiagnosis and misdiagnosis. Unlike previous studies emphasizing classification of known infections, our model is designed for early screening and de novo detection in routine imaging.

Transformer models have been used as classification models for medical images, including Swin-Transformer, Vision Transformer (ViT) and UNet Transformer *etc*. The study by Zhou et al. (2024) used the deep learning-based framework of the UNETR model with high accuracy and robustness in lung lesion segmentation and early ARDS prediction with good generalization ability and clinical applicability^22^. Smith et al. (2024) developed a Vision Transformer-based decision support system that accurately predicts the need for neurosurgical intervention using CT scans of acute traumatic brain injury. Based on this, in this study, we introduced the Swin Transformer Block module at the core of the feature processing (Wu et al., 2022), transformation stage and output link, which combines the shift window operation and the global attention mechanism to improve the model’s ability in local perception and overall information comprehension, and it has obvious advantages in CT diagnosis of spinal infection compared with other deep learning models (Du et al., 2024). Although the DL model demonstrated human-equivalent performance at the patient level, this task is relatively coarse and does not fully capture its clinical value. Slice-level detection of spinal infection is substantially more challenging and provides actionable information for lesion localization, treatment planning, and disease monitoring. Therefore, we prioritized slice-level analysis as the primary focus of this study.

The deep learning model in this study has good diagnostic efficiency for spinal infections, especially the sensitivity for lesion identification, which is much higher than that of radiologists. With the assistance of the model, radiologists significantly improved the diagnostic efficiency and reduced the diagnostic time, especially for the sensitivity of lesion diagnosis, with a good improvement. Due to the complexity of spinal infections, different parts of the infection, age, gender, and concomitant lesions may affect the diagnostic rate of the model. In this study, spinal epidural abscess and pathogen were factors that affected the diagnostic efficiency, probably because it is possible that these complications lead to significant differences in the signals of the surrounding tissues and the vertebral body, and that these differences affect the identification of the lesion. In addition, chest and abdominal CT slices also have good diagnostic performance. In summary, our model is the best-known artificial intelligence tool for spinal infection recognition *via* CT transections, which can assist radiologists to recognize spinal infections faster and more readily. The deep learning model proposed in this study demonstrated excellent diagnostic performance on non-contrast CT, suggesting its potential as a support tool for CT-based screening to enable early warning and triage in patients who are unable to undergo MRI. However, we emphasize that non-contrast CT is not intended to replace MRI, but rather serves as a complementary tool in specific clinical scenarios.

The interpretability of our model can be demonstrated by the Grad-CAM overlay, which shows that the model’s attention is focused on vertebral osteomyelitis (blue color), consistent with the radiologist’s diagnostic site (Fig. 2). In addition, the presence of an epidural abscess contributes to the model’s diagnosis, which indirectly suggests that the model meets the diagnostic criteria of human experts.

Beyond diagnostic performance, the proposed model holds promise for several real world clinical applications. First, it may serve as a triage and prioritization tool in emergency departments or resource limited settings, flagging suspected spinal infections on routine non contrast CT scans and thereby reducing the risk of incidental findings being overlooked (Crombé et al., 2024). Second, its slice level localization capability can directly support biopsy planning by highlighting the most representative level for percutaneous sampling, potentially increasing microbiological yield. However, as emphasized in a recent comprehensive review by Qu et al. (2022), the integration of deep learning into clinical workflows requires cautious interpretation. False positive predictions—inevitable in any high sensitivity system—may lead to unnecessary follow up imaging, patient anxiety, and increased healthcare costs. Therefore, radiologists must understand that this tool is designed as an assistive device, not a diagnostic replacement. Its outputs should always be corroborated with clinical history, laboratory findings, and, when indicated, MRI or histopathological confirmation. We believe that transparent reporting of model limitations and rigorous human AI interaction studies are essential next steps toward safe and effective clinical deployment.

Several limitations of this study should be acknowledged. First, the sample size, while comparable to many retrospective deep learning studies in rare diseases, remains modest. Although we employed strict patient-level splitting, extensive data augmentation, regularization, and—most importantly—independent external validation to mitigate overfitting, the possibility of residual bias cannot be entirely excluded. Larger multi-center studies with more diverse populations are warranted to further validate and refine our model.

Second, all training and internal validation data were obtained from a single institution, which may limit the diversity of imaging equipment and patient demographics, and thus affect the generalizability of our findings. To address this, we have already collected and evaluated an independent external validation cohort from a different institution using different CT scanners and protocols, which demonstrated consistently high performance (AUC: 0.989; sensitivity: 98.2%; specificity: 98.3%). Nevertheless, we acknowledge that this external cohort was still limited in size and geographic scope. Future work should therefore include large-scale, international, multi-center prospective registries to comprehensively assess model performance across varied populations, imaging platforms, and disease prevalences, and to support regulatory approval for clinical deployment.

Third, the current model performs binary classification (infection *vs.* non-infection). Extending this framework to multi-class differential diagnosis represents a critical next step (Mo et al., 2026). In particular, distinguishing tuberculous spondylodiscitis from pyogenic or brucellar infections is of paramount clinical importance, as these entities require fundamentally different antimicrobial and surgical treatment strategies. Preliminary evidence from our subgroup analysis suggests that the model’s diagnostic confidence varies by pathogen type, warranting dedicated investigation. We therefore plan to develop a multi-label classification model capable of identifying specific etiologies directly from non-contrast CT images.

Fourth, the methodological framework established in this study—combining YOLO-based vertebral segmentation with Swin Transformer classification—is not disease-specific. Adapting this pipeline to other spinal pathologies, such as metastatic disease, osteoporotic compression fractures, multiple myeloma, and ankylosing spondylitis, could accelerate the development of a comprehensive, multi-task AI system for spinal imaging. Such a system would be of substantial value in routine clinical practice, where differentiating infection from tumor or degeneration is a common diagnostic challenge.

Finally, while our study demonstrated that AI assistance significantly improves radiologist sensitivity and reduces reading time, this evaluation was conducted in an experimental, off-line setting. The true clinical impact of our model—on diagnostic turnaround time, inter-reader agreement, and, ultimately, patient outcomes such as time to appropriate antibiotic therapy or surgical intervention—remains to be determined. We therefore consider a prospective, pragmatic clinical trial embedded into real-world radiology workflows to be the highest priority for our future research. Such a trial will rigorously assess the feasibility, acceptability, and clinical utility of our model in authentic clinical environments.

## Conclusion

In conclusion, this deep learning system demonstrated excellent diagnostic performance in identifying spinal infections on non-contrast CT and significantly improved radiologists’ reading speed and efficiency. Within the confines of our dataset, the model achieved diagnostic accuracy approaching that of MRI, as reported in the literature (Qi et al., 2024). However, we emphatically caution that non-contrast CT is not intended to replace MRI, which remains the indisputable gold standard for spinal infection diagnosis due to its superior soft tissue contrast and assessment of neural compromise. If these findings are confirmed through large-scale, prospective multi-centre validation, this approach may offer a complementary screening tool for specific clinical scenarios, such as patients with contraindications to MRI, emergency triage, or resource-limited settings where MRI access is constrained. Our method may ultimately facilitate earlier detection and treatment of spinal infections in these contexts and help reduce incidental underdiagnosis on routine CT.

## Supplemental Information

10.7717/peerj.21340/supp-1 Supplemental Information 1 Confusion matrices for the internal validation set (per-axial CT slice) Rows represent true labels and columns represent predicted labels (0: negative, 1: positive). Each confusion matrix displays the number of true positive (TP), false positive (FP), false negative (FN), and true negative (TN) predictions on a per-slice basis. Color intensity reflects the normalized proportion of predictions (row-wise normalization). (A) Deep learning model (standalone). (B) Radiologist 1 without DL assistance. (C) Radiologist 2 without DL assistance. (D) Radiologist 1 with DL assistance. (E) Radiologist 2 with DL assistance. Abbreviations: DL, deep learning; TN, true negative; FP, false positive; FN, false negative; TP, true positive.

10.7717/peerj.21340/supp-2 Supplemental Information 2 Confusion matrices for the external validation cohort (per-axial CT slice) Rows represent true labels and columns represent predicted labels (0: negative, 1: positive). Each confusion matrix presents the raw counts of TP, FP, FN, and TN predictions at the slice level. Color mapping is scaled to the maximum count in each matrix for visual clarity. (A) Deep learning model (standalone). (B) Radiologist 1 without DL assistance. (C) Radiologist 2 without DL assistance. (D) Radiologist 1 with DL assistance. (E) Radiologist 2 with DL assistance. Abbreviations: DL, deep learning; TN, true negative; FP, false positive; FN, false negative; TP, true positive.

10.7717/peerj.21340/supp-3 Supplemental Information 3 Patient-level confusion matrices for the combined internal validation and non-infected control cohort Rows represent true labels and columns represent predicted labels (0: negative, 1: positive). A patient was classified as positive if at least one axial slice from the entire CT series yielded a model probability ≥ 0.5; otherwise, negative. Each confusion matrix presents the raw counts of true positive (TP), false positive (FP), false negative (FN), and true negative (TN) predictions at the patient level. Color intensity is scaled to the maximum count in each matrix for visual clarity. (A) Deep learning model (standalone). (B) Radiologist 1 without DL assistance. (C) Radiologist 2 without DL assistance. Abbreviations: DL, deep learning; TP, true positive; FP, false positive; FN, false negative; TN, true negative.

10.7717/peerj.21340/supp-4 Supplemental Information 4 Distribution of CT slices per patient

10.7717/peerj.21340/supp-5 Supplemental Information 5 Patient-level diagnostic performance of the deep learning model and radiologists

10.7717/peerj.21340/supp-6 Supplemental Information 6 Supplementary materials: patient-level analysis

10.7717/peerj.21340/supp-7 Supplemental Information 7 Raw data

10.7717/peerj.21340/supp-8 Supplemental Information 8 Model code

10.7717/peerj.21340/supp-9 Supplemental Information 9 CLAIM checklist

10.7717/peerj.21340/supp-10 Supplemental Information 10 STROBE checklist

10.7717/peerj.21340/supp-11 Supplemental Information 11 Codebook

## Additional Information and Declarations

### Data Availability

The following information was supplied regarding data availability: The raw data and code are available in the Supplemental Files.
