# Supplementary figures and images for: A transformer-based deep learning algorithm for diagnosing spinal infections on axial non-contrast computed tomography images: a dual-center retrospective study
Source: PeerJ. 2026 Jun 11;14:e21340. doi: 10.7717/peerj.21340 (PMC13264972; doi:10.7717/peerj.21340)

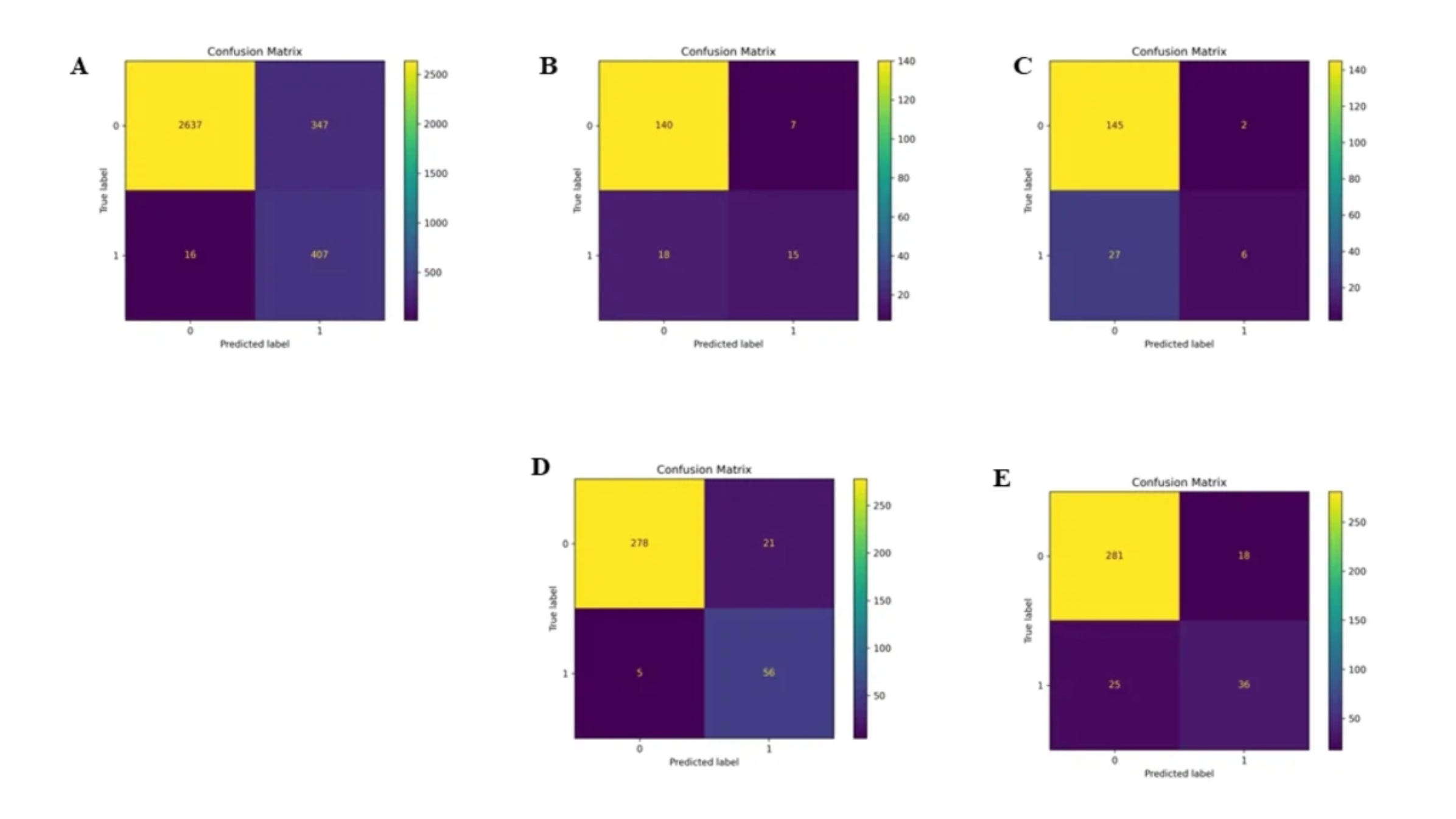

Supplement: Supplemental Information 1 — Rows represent true labels and columns represent predicted labels (0: negative, 1: positive). Each confusion matrix displays the number of true positive (TP), false positive (FP), false negative (FN), and true negative (TN) predictions on a per-slice basis. Color intensity reflects the normalized proportion of predictions (row-wise normalization). (A) Deep learning model (standalone). (B) Radiologist 1 without DL assistance. (C) Radiologist 2 without DL assistance. (D) Radiologist 1 with DL assistance. (E) Radiologist 2 with DL assistance. Abbreviations: DL, deep learning; TN, true negative; FP, false positive; FN, false negative; TP, true positive. [file peerj-14-21340-s001.png]

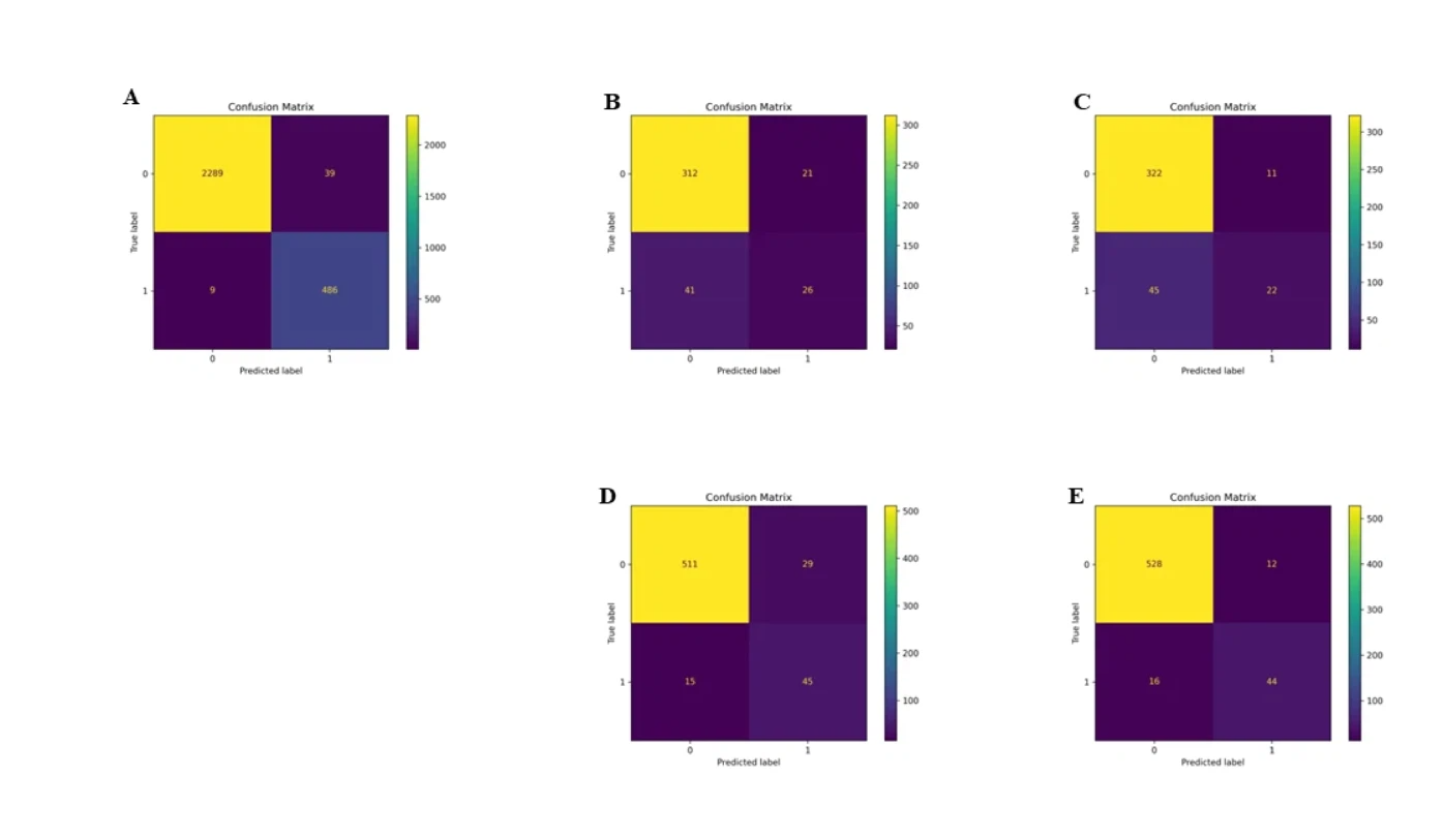

Supplement: Supplemental Information 2 — Rows represent true labels and columns represent predicted labels (0: negative, 1: positive). Each confusion matrix presents the raw counts of TP, FP, FN, and TN predictions at the slice level. Color mapping is scaled to the maximum count in each matrix for visual clarity. (A) Deep learning model (standalone). (B) Radiologist 1 without DL assistance. (C) Radiologist 2 without DL assistance. (D) Radiologist 1 with DL assistance. (E) Radiologist 2 with DL assistance. Abbreviations: DL, deep learning; TN, true negative; FP, false positive; FN, false negative; TP, true positive. [file peerj-14-21340-s002.png]

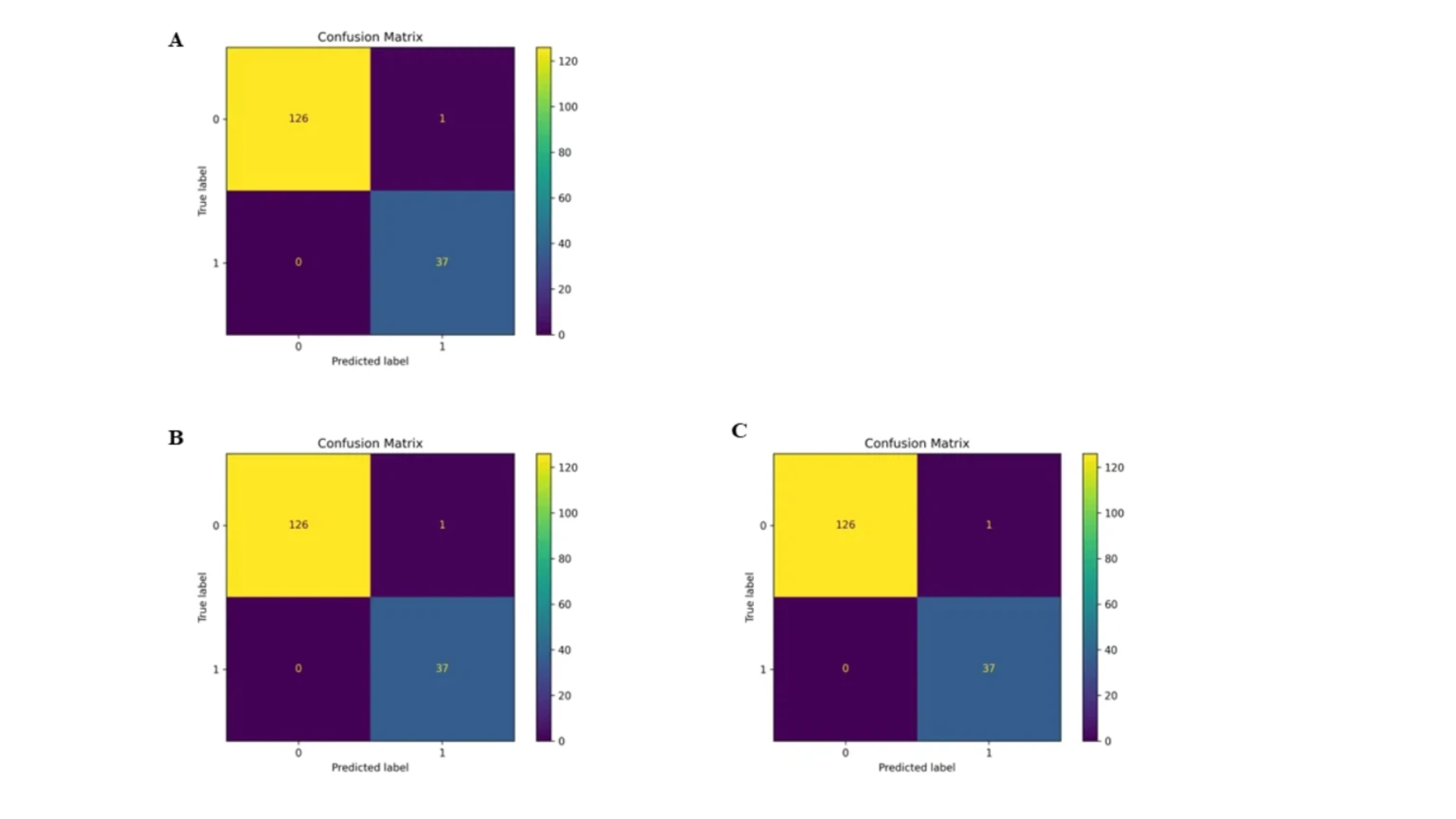

Supplement: Supplemental Information 3 — Rows represent true labels and columns represent predicted labels (0: negative, 1: positive). A patient was classified as positive if at least one axial slice from the entire CT series yielded a model probability ≥ 0.5; otherwise, negative. Each confusion matrix presents the raw counts of true positive (TP), false positive (FP), false negative (FN), and true negative (TN) predictions at the patient level. Color intensity is scaled to the maximum count in each matrix for visual clarity. (A) Deep learning model (standalone). (B) Radiologist 1 without DL assistance. (C) Radiologist 2 without DL assistance. Abbreviations: DL, deep learning; TP, true positive; FP, false positive; FN, false negative; TN, true negative. [file peerj-14-21340-s003.png]
