# Supplementary material for: A transformer-based deep learning algorithm for diagnosing spinal infections on axial non-contrast computed tomography images: a dual-center retrospective study
Source: PeerJ. 2026 Jun 11;14:e21340. doi: 10.7717/peerj.21340 (PMC13264972; doi:10.7717/peerj.21340)
Supplement: Supplemental Information 4 [file peerj-14-21340-s004.docx]

**Supplementary Table 1. Distribution of CT slices per patient**

| Cohort | No. of Patients | Mean | SD* | Min | Max |
| --- | --- | --- | --- | --- | --- |
| Internal Validation | 127 | 101 | 6 | 52 | 181 |
| External Validation | 30 | 92 | 5.5 | 46 | 167 |

*SD: standard deviation (calculated as the square root of variance).
