# Supplementary material for: A transformer-based deep learning algorithm for diagnosing spinal infections on axial non-contrast computed tomography images: a dual-center retrospective study
Source: PeerJ. 2026 Jun 11;14:e21340. doi: 10.7717/peerj.21340 (PMC13264972; doi:10.7717/peerj.21340)
Supplement: Supplemental Information 5 [file peerj-14-21340-s005.docx]

**Supplementary Table 2. Patient‑Level Diagnostic Performance of the Deep Learning Model and Radiologists**

| **Metric** | **DL Model** | **Radiologist 1** | **Radiologist 2** | *P value (DL vs. R1)** | *P value (DL vs. R2)** |
| --- | --- | --- | --- | --- | --- |
| **True Positives (TP)** | 37 | 37 | 37 | – | – |
| **False Positives (FP)** | 1 | 1 | 1 | – | – |
| **False Negatives (FN)** | 0 | 0 | 0 | – | – |
| **True Negatives (TN)** | 126 | 126 | 126 | – | – |
| **Sensitivity (%)** | 100 (90.5–100) | 100 (90.5–100) | 100 (90.5–100) | ＞0.05 | ＞0.05 |
| **Specificity (%)** | 99.2 (95.7–99.9) | 99.2 (95.7–99.9) | 99.2 (95.7–99.9) | ＞0.05 | ＞0.05 |
| **Accuracy (%)** | 99.4 (96.6–99.9) | 99.4 (96.6–99.9) | 99.4 (96.6–99.9) | ＞0.05 | ＞0.05 |
| **PPV (%)** | 97.4 (86.2–99.9) | 97.4 (86.2–99.9) | 97.4 (86.2–99.9) | ＞0.05 | ＞0.05 |
| **NPV (%)** | 100 (97.1–100) | 100 (97.1–100) | 100 (97.1–100) | ＞0.05 | ＞0.05 |
| **AUC** | 0.996 (0.989–1.000) | 0.996 (0.989–1.000) | 0.996 (0.989–1.000) | ＞0.05 | ＞0.05 |
| **F1‑score** | 0.987 | 0.987 | 0.987 | ＞0.05 | ＞0.05 |

*Data in parentheses are 95% confidence intervals (CIs). Confidence intervals for proportions were calculated using the Clopper–Pearson exact method; CIs for AUC were derived using DeLong’s method.*
*† P values were calculated using McNemar’s test for paired binary classifications (sensitivity, specificity, accuracy, PPV, NPV) and DeLong’s test for AUC comparison. All comparisons yielded non-significant differences (P > 0.05), indicating human‑equivalent diagnostic performance at the patient level.*
