# Supplementary material for: A transformer-based deep learning algorithm for diagnosing spinal infections on axial non-contrast computed tomography images: a dual-center retrospective study
Source: PeerJ. 2026 Jun 11;14:e21340. doi: 10.7717/peerj.21340 (PMC13264972; doi:10.7717/peerj.21340)
Supplement: Supplemental Information 6 [file peerj-14-21340-s006.docx]

**Supplementary Information: Detailed Patient‑Level Diagnostic Performance** Analysis

To complement the slice‑level evaluation presented in the main manuscript, we conducted a comprehensive patient‑level diagnostic performance analysis. This analysis serves two purposes: (i) to assess the clinical utility of the deep learning model as a patient‑screening tool, and (ii) to provide a direct comparison with radiologists’ patient‑level diagnostic decisions. The results are summarized in Supplementary Table S1 and described in detail below.

**1. Study Cohort for Patient‑Level Analysis**

The patient‑level evaluation cohort comprised 164 independent patients, including:

- 37 infected patients from the internal validation set (confirmed by postoperative pathology or etiological testing);
- 127 non‑infected control patients who underwent non‑contrast CT for non‑spinal indications during the same period and had no evidence of spinal infection confirmed by ≥6 months of clinical follow‑up.

Importantly, none of these 127 control patients were used during model training or internal validation, ensuring an unbiased assessment of specificity and negative predictive value. This cohort was assembled to reflect the real‑world screening scenario, where the prevalence of spinal infection is low and the model must correctly rule out a large number of truly negative individuals.

**2. Patient‑Level Classification Rule**

A patient was classified as positive for spinal infection if at least one axial slice from their entire non‑contrast CT series yielded a model prediction probability ≥ 0.5 (the same threshold used for slice‑level classification). Otherwise, the patient was classified as negative. This “any‑slice‑positive” rule directly mirrors clinical practice: a patient is diagnosed with spinal infection if any imaging evidence is identified, regardless of its extent.

**3. Patient‑Level Confusion Matrix and Performance Metrics**

The patient‑level confusion matrix and derived performance metrics are presented Supplementary Table S2.

Statistical comparison with radiologists:
McNemar’s test was applied to compare the patient‑level misclassification counts between the DL model and each of the two radiologists (based on their independent interpretations of the same 164‑patient cohort). The results showed no statistically significant difference (DL vs. Radiologist 1: P > 0.05; DL vs. Radiologist 2: P > 0.05), indicating that the model achieved human‑equivalent diagnostic performance at the patient level.

**4. Interpretation of the Single False Positive Case**

The sole false positive case (FP = 1) involved a 72‑year‑old male patient with severe degenerative disc disease and Modic type II endplate changes. The model erroneously assigned a probability of 0.87 to a single slice showing endplate irregularity. On retrospective review, both radiologists agreed that the imaging findings were equivocal and could not definitively exclude early infection. This case highlights the inherent difficulty of distinguishing infection from degenerative inflammation, even for expert readers, and was therefore considered an acceptable trade‑off given the model’s perfect sensitivity.

**5. Rationale for Emphasizing Slice‑Level Analysis in the Main Manuscript**

While the patient‑level results demonstrate that the model is an excellent screening tool (100% sensitivity, 99.2% specificity), this task is relatively coarse and does not fully capture the model’s unique clinical value. Both the DL model and radiologists achieved near‑ceiling performance at the patient level, leaving limited room for discrimination.

In contrast, slice‑level detection of spinal infection is substantially more challenging due to:

- High class imbalance: positive slices constitute only ~11% of all slices;
- Subtle and focal imaging findings: infection may involve only a single vertebral level or a small paravertebral abscess;
- Clinical actionability: precise lesion localization is essential for guiding biopsy, determining the extent of surgical debridement, and monitoring treatment response.

Therefore, the main manuscript focuses on slice‑level metrics (AUC, sensitivity, specificity, PPV, NPV, F1‑score) because they:

1. Reflect the true discriminative power of the model under realistic, high‑complexity conditions;
2. Enable direct comparison with radiologists at the most granular diagnostic level;
3. Demonstrate the model’s added value as a localization tool rather than merely a binary screener.

The patient‑level analysis is presented here as complementary evidence that the model’s excellent slice‑level performance translates into robust clinical decision‑making at the patient level.

**6. Availability of Slice‑Level Confusion Matrices**

For complete transparency, the full slice‑level confusion matrices for both the internal and external validation cohorts are provided in Supplementary Figure S1. These matrices contain the exact counts of true positives, false positives, true negatives, and false negatives used to derive the performance metrics reported in Tables 2 and 3 of the main text.

**Conclusion:** The patient‑level analysis confirms that our deep learning model achieves human‑equivalent diagnostic accuracy when used as a screening tool for spinal infection. The single false positive case is clinically acceptable given the model’s perfect sensitivity. Nevertheless, the primary contribution of this work lies in slice‑level lesion detection, which addresses a more clinically demanding task and provides actionable information for patient management. We believe this two‑level evaluation framework offers a complete and transparent assessment of the model’s capabilities.
