# Supplementary material for: A transformer-based deep learning algorithm for diagnosing spinal infections on axial non-contrast computed tomography images: a dual-center retrospective study
Source: PeerJ. 2026 Jun 11;14:e21340. doi: 10.7717/peerj.21340 (PMC13264972; doi:10.7717/peerj.21340)
Supplement: Supplemental Information 9 [file peerj-14-21340-s009.docx]

Below is a **CLAIM (Checklist for Artificial Intelligence in Medical Imaging)** template based strictly on the table from Mongan et al., *Radiology: Artificial Intelligence* 2020.
You can use it to document where each item is addressed in your manuscript or to indicate that it is not applicable.

**CLAIM Checklist**

| Item | Description | Location / Notes *(to be filled by author)* |
| --- | --- | --- |
| 1 | Identification as a study of AI methodology, specifying the category of technology used (e.g., deep learning) | Title; Abstract; Introduction (Swin Transformer, deep learning) |
| 2 | Structured summary of study design, methods, results, and conclusions | Abstract |
| 3 | Scientific and clinical background, including the intended use and clinical role of the AI approach | Introduction (paragraphs 1–5); Discussion (paragraphs 1, 8) |
| 4 | Study objectives and hypotheses | introduction (last paragraph); Methods (Study Design and Participants) |
| 5 | Prospective or retrospective study | Abstract (Methods); Methods (Study Design and Participants)/ retrospective study |
| 6 | Study goal, such as model creation, exploratory study, feasibility study, noninferiority trial | Introduction (last paragraph); Methods (Deep Learning Model Development) |
| 7 | Data sources | Methods (Study Design and Participants); Methods (Imaging acquisition) |
| 8 | Eligibility criteria: how, where, and when potentially eligible participants or studies were identified (e.g., symptoms, results from previous tests, inclusion in registry, patient‑care setting, location, dates) | Methods (Study Design and Participants); Figure 1 |
| 9 | Data preprocessing steps | Methods (Image Annotation and Slice Classification); Methods (Deep Learning Model Development) |
| 10 | Selection of data subsets, if applicable | Methods (Study Design and Participants) — training/internal validation split; not applicable for subset selection beyond partitioning |
| 11 | Definitions of data elements, with references to common data elements | Not applicable (no common data elements referenced) |
| 12 | De‑identification methods | Methods (Imaging acquisition) — “anonymized prior to analysis” |
| 13 | How missing data were handled | exclusion criteria removed incomplete cases |
| 14 | Definition of ground truth reference standard, in sufficient detail to allow replication | Methods (Image Annotation and Slice Classification) — manual slice-level segmentation under radiologist supervision |
| 15 | Rationale for choosing the reference standard (if alternatives exist) | Methods (Image Annotation and Slice Classification); gold standard: postoperative pathology or etiological testing |
| 16 | Source of ground truth annotations; qualifications and preparation of annotators | Methods (Image Annotation and Slice Classification) — two musculoskeletal radiologists (>10 years experience) |
| 17 | Annotation tools | Methods (Image Annotation and Slice Classification) — “dedicated annotation platform” |
| 18 | Measurement of inter‑ and intrarater variability; methods to mitigate variability and/or resolve discrepancies | Methods (Image Annotation and Slice Classification) — independent review by radiologist, consensus resolution |
| 19 | Intended sample size and how it was determined | Methods (Study Design and Participants) — enrollment based on eligibility; not powered a priori |
| 20 | How data were assigned to partitions; specify proportions | Methods (Study Design and Participants) — 7:3 random split, computer-generated |
| 21 | Level at which partitions are disjoint (e.g., image, study, patient, institution) | Methods (Study Design and Participants) — patient-level splitting; external cohort from separate institution |
| 22 | Detailed description of model, including inputs, outputs, all intermediate layers and connections | Methods (Deep Learning Model Development); Figure 2 — YOLO segmentation + Swin Transformer |
| 23 | Software libraries, frameworks, and packages | Python |
| 24 | Initialization of model parameters (e.g., randomization, transfer learning) | Methods (Deep Learning Model Development) |
| 25 | Details of training approach, including data augmentation, hyperparameters, number of models trained | Methods (Deep Learning Model Development) — data augmentation, oversampling, L2 weight decay (λ = 5×10⁻⁴), binary cross-entropy loss |
| 26 | Method of selecting the final model | Methods (Multiple comparisons of deep learning methods) — Swin-Transformer selected based on highest efficacy in training group |
| 27 | Ensembling techniques, if applicable | Not applicable |
| 28 | Metrics of model performance | Methods (Statistical Analysis); Results — AUC, AUPRC, sensitivity, specificity, accuracy, PPV, NPV, F1-score |
| 29 | Statistical measures of significance and uncertainty (e.g., confidence intervals) | Methods (Statistical Analysis) — DeLong method for AUC CIs, bootstrap for AUPRC, Clopper-Pearson for binary metrics; 95% CIs reported in tables |
| 30 | Robustness or sensitivity analysis | Results (Subgroup Analysis by CT Scan Type) — stratified by CT protocol; Table 10 (factors affecting performance) |
| 31 | Methods for explainability or interpretability (e.g., saliency maps) and how they were validated | Methods (Deep Learning Model Development); Results (Interpretability of the model) — Grad-CAM, visual alignment with radiologist features |
| 32 | Validation or testing on external data | Methods (Study Design and Participants) — external cohort from Henan Medical University (n=30); Results (external validation tables) |
| 33 | Flow of participants or cases, using a diagram to indicate inclusion and exclusion | Figure 1; Methods (Study Design and Participants) — patient screening process |
| 34 | Demographic and clinical characteristics of cases in each partition | Table 1; Results (Patient Characteristic) |
| 35 | Performance metrics for optimal model(s) on all data partitions | Tables 2, 3, 4, 5; Results (Diagnostic performance, AI assistance) |
| 36 | Estimates of diagnostic accuracy and their precision (such as 95% confidence intervals) | Tables 2–5 (95% CIs reported); Methods (Statistical Analysis) |
| 37 | Failure analysis of incorrectly classified cases | Results (Factors affect the diagnostic efficiency of deep learning) — SEA and pathogen type identified as influencing factors; no dedicated failure analysis section |
| 38 | Study limitations, including potential bias, statistical uncertainty, and generalizability | Discussion (Limitations) |
| 39 | Implications for practice, including the intended use and/or clinical role | Discussion (paragraphs 8–9); Conclusio |
| 40 | Registration number and name of registry | Not applicable (retrospective study, not a clinical trial) |
| 41 | Where the full study protocol can be accessed | Not applicable |
| 42 | Sources of funding and other support; role of funders | title page |

**Instructions**

- For each item, indicate the section/page/line number in your manuscript where the information appears, or note “N/A” if not applicable.
