# Supplementary material for: A transformer-based deep learning algorithm for diagnosing spinal infections on axial non-contrast computed tomography images: a dual-center retrospective study
Source: PeerJ. 2026 Jun 11;14:e21340. doi: 10.7717/peerj.21340 (PMC13264972; doi:10.7717/peerj.21340)
Supplement: Supplemental Information 10 [file peerj-14-21340-s010.docx]

STROBE Statement—checklist of items that should be included in reports of observational studies

|  | Item No. | Recommendation | Page  No. | Relevant text from manuscript |
| --- | --- | --- | --- | --- |
| **Title and abstract** | 1 | (*a*) Indicate the study’s design with a commonly used term in the title or the abstract | 1 | In this retrospective study, non-contrast CT data from 127 patients diagnosed with spinal infections between 2020 and 2023 were used. |
|  |  | (*b*) Provide in the abstract an informative and balanced summary of what was done and what was found | 1 | The transformer-based deep learning model effectively diagnosed spinal infections using non-contrast CT images, surpassing radiologists in performance. It enhanced radiologists' diagnostic efficiency and accuracy. |
| Introduction | | | |  |
| Background/rationale | 2 | Explain the scientific background and rationale for the investigation being reported | 2-3 | Page2 Line1-page3Line12 |
| Objectives | 3 | State specific objectives, including any prespecified hypotheses | 3-4 | Page3 Line13-page4Line5 |
| Methods | | | |  |
| Study design | 4 | Present key elements of study design early in the paper | 4 | We retrospectively reviewed the records of patients with spondylodiscitis from November 2020 to August 2023 in the First Affiliated Hospital, Zhejiang University of Medicine. |
| Setting | 5 | Describe the setting, locations, and relevant dates, including periods of recruitment, exposure, follow-up, and data collection | 4 | We retrospectively reviewed the records of patients with spondylodiscitis from November 2020 to August 2023 in the First Affiliated Hospital, Zhejiang University of Medicine.127 patients with a confirmed diagnosis of spinal infection were included in the study. Data collection was conducted from March 2024 to July 2024, which included identifying eligible patients and retrieving their non-contrast CT scans and basic patient information from the Picture Archiving and Communication System (PACS). |
| Participants | 6 | (*a*) *Cohort study*—Give the eligibility criteria, and the sources and methods of selection of participants. Describe methods of follow-up  *Case-control study*—Give the eligibility criteria, and the sources and methods of case ascertainment and control selection. Give the rationale for the choice of cases and controls  *Cross-sectional study*—Give the eligibility criteria, and the sources and methods of selection of participants | 4 | We retrospectively reviewed the records of patients with spondylodiscitis from November 2020 to August 2023 in the First Affiliated Hospital, Zhejiang University of Medicine. The inclusion criteria were a definitive diagnosis of spinal infection based on postoperative pathological examination or pathogenetic testing, and represented preoperative non-contrast CT scans, and complete clinical data. The exclusion criteria were: 1. Unclear postoperative pathological diagnosis; 2. Concurrent tumors or other immune-related diseases; 3. Incomplete or unclear imaging data; 4. Incomplete clinical data. |
|  |  | (*b*) *Cohort study*—For matched studies, give matching criteria and number of exposed and unexposed  *Case-control study*—For matched studies, give matching criteria and the number of controls per case |  |  |
| Variables | 7 | Clearly define all outcomes, exposures, predictors, potential confounders, and effect modifiers. Give diagnostic criteria, if applicable | 4-5 | spinal infection based on postoperative pathological examination or pathogenetic testing. Pathogen identification was confirmed through standard bacterial culture or next-generation sequencing (NGS) of specimens. |
| Data sources/ measurement | 8* | For each variable of interest, give sources of data and details of methods of assessment (measurement). Describe comparability of assessment methods if there is more than one group | *5* | For the retrospective recruitment of patients into the training and test sets, the Institutional Review Board waived the requirement for informed consent. as shown in Figure 1. |
| Bias | 9 | Describe any efforts to address potential sources of bias | 5 | All CT scans were performed with slice thickness ≤1.25 mm (range 0.625-1.25 mm). All CT data were obtained without the use of contrast examination and sagittal reconstruction. All the selected images were normalized to window width 300-500HU, window position 40-60HU to optimize soft tissue contrast and resized to 512 × 512 pixels and uploaded to pair. A radiologic technician, under the general supervision of two board-certified musculoskeletal radiologists (with 10 years of experience), manually mapped the area of each spinal infection, including the size, location, and shape of the segmental lesion. |
| Study size | 10 | Explain how the study size was arrived at | 12 | While formal sample size calculation was not performed a priori due to the exploratory and retrospective nature of this deep learning diagnostic study, the cohort of 127 patients provided a robust foundation for model development and preliminary validation. The dataset was partitioned into a training set (n=89, 70%) for model optimization and an independent test set (n=38, 30%) for performance evaluation. This split aligns with common practices in machine learning studies aiming to balance model training needs and statistical assessment reliability. Post-hoc power analysis confirmed that the achieved sensitivity (94.5%) and specificity (89.9%) on the test set had narrow confidence intervals, indicating sufficient precision for initial algorithm validation. However, larger multi-center samples are recommended in future work to enhance generalizability and further reduce potential overfitting. |

Continued on next page

| Quantitative variables | 11 | Explain how quantitative variables were handled in the analyses. If applicable, describe which groupings were chosen and why | 4,6-7 | The patients were randomly assigned to the training and test sets in an approximately 7:3 ratio. Continuous data are expressed as mean and standard deviation, while categorical data are expressed as counts and percentages. Then, the performance outcomes were derived from an area under the receiver operating characteristic curve (AUROC) for each human reader, with and without AI assistance, and also for the stand-alone AI reader. In addition, sensitivity(sen), specificity(spe), accuracy(acc), and error rate values were calculated and compared for each reader using the McNemar test, and Chi -square test was used to calculate the positive predictive value (PPV) and negative predictive value (NPV). |
| --- | --- | --- | --- | --- |
| Statistical methods | 12 | (*a*) Describe all statistical methods, including those used to control for confounding | 6-7 | Page6line18-page7line6 |
|  |  | (*b*) Describe any methods used to examine subgroups and interactions | 6-7 | Page6line18-page7line6 |
|  |  | (*c*) Explain how missing data were addressed | 6-7 | Page6line18-page7line6 |
|  |  | (*d*) *Cohort study*—If applicable, explain how loss to follow-up was addressed  *Case-control study*—If applicable, explain how matching of cases and controls was addressed  *Cross-sectional study*—If applicable, describe analytical methods taking account of sampling strategy | 6-7 | Page6line18-page7line6 |
|  |  | (*e*) Describe any sensitivity analyses | 6-7 | Page6line18-page7line6 |
| Results | | | | |
| Participants | 13* | (a) Report numbers of individuals at each stage of study—eg numbers potentially eligible, examined for eligibility, confirmed eligible, included in the study, completing follow-up, and analysed | 7 | Line9-line17 |
|  |  | (b) Give reasons for non-participation at each stage | 7 | Line9-line17 |
|  |  | (c) Consider use of a flow diagram | 7 | Figure1 |
| Descriptive data | 14* | (a) Give characteristics of study participants (eg demographic, clinical, social) and information on exposures and potential confounders | 7 | Line9-line17 |
|  |  | (b) Indicate number of participants with missing data for each variable of interest | 7 | Line9-line17 |
|  |  | (c) *Cohort study*—Summarise follow-up time (eg, average and total amount) | 7 | Line9-line17 |
| Outcome data | 15* | *Cohort study*—Report numbers of outcome events or summary measures over time |  |  |
|  |  | *Case-control study—*Report numbers in each exposure category, or summary measures of exposure | *7* | *Table 1* |
|  |  | *Cross-sectional study—*Report numbers of outcome events or summary measures |  |  |
| Main results | 16 | (*a*) Give unadjusted estimates and, if applicable, confounder-adjusted estimates and their precision (eg, 95% confidence interval). Make clear which confounders were adjusted for and why they were included | 9-10 | *Table 1*  Different factors may affect the diagnostic efficacy of deep learning. Therefore, we analyzed the spinal segments, the image type, the presence or absence of concomitant spinal epidural abscess, pathogen, sex, and age for comparison. We found that the presence or absence of spinal epidural abscess and pathogen, influences the diagnostic outcome(P<0.001) (see Table 5). |
|  |  | (*b*) Report category boundaries when continuous variables were categorized | 9-10 | *Table 1*  Different factors may affect the diagnostic efficacy of deep learning. Therefore, we analyzed the spinal segments, the image type, the presence or absence of concomitant spinal epidural abscess, pathogen, sex, and age for comparison. We found that the presence or absence of spinal epidural abscess and pathogen, influences the diagnostic outcome(P<0.001) (see Table 5). |
|  |  | (*c*) If relevant, consider translating estimates of relative risk into absolute risk for a meaningful time period | 9-10 | *Table 1* |

Continued on next page

| Other analyses | 17 | Report other analyses done—eg analyses of subgroups and interactions, and sensitivity analyses | 7-10 |  |
| --- | --- | --- | --- | --- |
| Discussion | | | | |
| Key results | 18 | Summarise key results with reference to study objectives | 10-12 | The deep learning model in this study has good diagnostic efficiency for spinal infections, especially the sensitivity for lesion identification, which is much higher than that of radiologists. With the assistance of the model, radiologists significantly improved the diagnostic efficiency and reduced the diagnostic time, especially for the sensitivity of lesion diagnosis, with a good improvement. Due to the complexity of spinal infections, different parts of the infection, age, gender, and concomitant lesions may affect the diagnostic rate of the model. In this study, spinal epidural abscess and pathogen were factors that affected the diagnostic efficiency |
| Limitations | 19 | Discuss limitations of the study, taking into account sources of potential bias or imprecision. Discuss both direction and magnitude of any potential bias | 12-13 | Our study has several potential limitations. First, the sample size, though aligned with common practices in retrospective deep-learning studies, remains moderate due to the inherent rarity of spinal infections. Although a formal sample size calculation was not performed a priori given the exploratory nature of this diagnostic study, the cohort of 127 patients was partitioned in a 70:30 ratio into training (n=89) and independent test sets (n=38), providing a reasonable foundation for model development and initial validation. Post-hoc analysis indicated that the model achieved high sensitivity (94.5%) and specificity (89.9%) with narrow confidence intervals, supporting the precision of these performance estimates. Nevertheless, the limited sample may affect generalizability, and future multi-center studies with larger cohorts are warranted to enhance robustness and reduce overfitting. |
| Interpretation | 20 | Give a cautious overall interpretation of results considering objectives, limitations, multiplicity of analyses, results from similar studies, and other relevant evidence | 10-12 | In summary, our model is the best-known artificial intelligence tool for spinal infection recognition via CT transections, which can assist radiologists to recognize spinal infections faster and more readily. In this study, spinal epidural abscess and pathogen were factors that affected the diagnostic efficiency, probably because it is possible that these complications lead to significant differences in the signals of the surrounding tissues and the vertebral body, and that these differences affect the identification of the lesion. In addition, chest and abdominal CT slices also have good diagnostic performance. In summary, our model is the best-known artificial intelligence tool for spinal infection recognition via CT transections, which can assist radiologists to recognize spinal infections faster and more readily. |
| Generalisability | 21 | Discuss the generalisability (external validity) of the study results | 13-14 | In conclusion, the deep learning system showed excellent diagnostic performance in identifying spinal infections on non-contrast CT and significantly improved radiologists' reading speed and efficiency. This suggests non-enhanced CT could become a routine diagnostic method for spinal infections, comparable to MRI, potentially reducing screening costs and enhancing diagnostic efficiency in emergency patients. Our method may facilitate the early identification and treatment of spinal infections in ordinary clinical practice and reduce incidental underdiagnosis. |
| Other information | |  | | |
| Funding | 22 | Give the source of funding and the role of the funders for the present study and, if applicable, for the original study on which the present article is based | 14 | This research was supported by Zhejiang Provincial Natural Science Foundation of China under Grant No. LQ22H100003 and the medical Health Science and Technology Project of Zhejiang Provincial Health Commission No.2020KY132. |

*Give information separately for cases and controls in case-control studies and, if applicable, for exposed and unexposed groups in cohort and cross-sectional studies.

**Note:** An Explanation and Elaboration article discusses each checklist item and gives methodological background and published examples of transparent reporting. The STROBE checklist is best used in conjunction with this article (freely available on the Web sites of PLoS Medicine at http://www.plosmedicine.org/, Annals of Internal Medicine at http://www.annals.org/, and Epidemiology at http://www.epidem.com/). Information on the STROBE Initiative is available at www.strobe-statement.org.
